# A genome-wide screen identifies SCAI as a modulator of the UV-induced replicative stress response

**DOI:** 10.1371/journal.pbio.3001543

**Published:** 2022-10-10

**Authors:** Jean-François Lemay, Edlie St-Hilaire, Daryl A. Ronato, Yuandi Gao, François Bélanger, Sari Gezzar-Dandashi, Aimé Boris Kimenyi Ishimwe, Christina Sawchyn, Dominique Lévesque, Mary McQuaid, François-Michel Boisvert, Frédérick A. Mallette, Jean-Yves Masson, Elliot A. Drobetsky, Hugo Wurtele

**Affiliations:** 1 Centre de recherche, de l’Hôpital Maisonneuve-Rosemont, Montréal, Québec, Canada; 2 Genome Stability Laboratory, CHU de Québec Research Center, Oncology Division; Department of Molecular Biology, Medical Biochemistry and Pathology; Laval University Cancer Research Center, Québec City, Québec, Canada; 3 Molecular Biology Program, Université de Montréal, Montréal, Québec, Canada; 4 Department of Biochemistry and Molecular Medicine, Université de Montréal, Montréal, Québec, Canada; 5 Department of Immunology and Cell Biology, Université de Sherbrooke, Sherbrooke, Québec, Canada; 6 Department of Medicine, Université de Montréal, Montréal, Québec, Canada; The Univ. of Texas.at Austin, UNITED STATES

## Abstract

Helix-destabilizing DNA lesions induced by environmental mutagens such as UV light cause genomic instability by strongly blocking the progression of DNA replication forks (RFs). At blocked RF, single-stranded DNA (ssDNA) accumulates and is rapidly bound by Replication Protein A (RPA) complexes. Such stretches of RPA-ssDNA constitute platforms for recruitment/activation of critical factors that promote DNA synthesis restart. However, during periods of severe replicative stress, RPA availability may become limiting due to inordinate sequestration of this multifunctional complex on ssDNA, thereby negatively impacting multiple vital RPA-dependent processes. Here, we performed a genome-wide screen to identify factors that restrict the accumulation of RPA-ssDNA during UV-induced replicative stress. While this approach revealed some expected “hits” acting in pathways such as nucleotide excision repair, translesion DNA synthesis, and the intra-S phase checkpoint, it also identified SCAI, whose role in the replicative stress response was previously unappreciated. Upon UV exposure, SCAI knock-down caused elevated accumulation of RPA-ssDNA during S phase, accompanied by reduced cell survival and compromised RF progression. These effects were independent of the previously reported role of SCAI in 53BP1-dependent DNA double-strand break repair. We also found that SCAI is recruited to UV-damaged chromatin and that its depletion promotes nascent DNA degradation at stalled RF. Finally, we (i) provide evidence that EXO1 is the major nuclease underlying ssDNA formation and DNA replication defects in SCAI knockout cells and, consistent with this, (ii) demonstrate that SCAI inhibits EXO1 activity on a ssDNA gap in vitro. Taken together, our data establish SCAI as a novel regulator of the UV-induced replicative stress response in human cells.

## Abstract 2

This study uses a genome-wide CRISPR-Cas9 screen to reveal new factors that influence the accumulation of Replication Protein A on DNA after UV irradiation; among these, the SCAI protein was found to bind and protect post-replicative single-stranded DNA gaps against extension by nucleases.

## Introduction

A variety of ubiquitous environmental genotoxins and chemotherapeutic drugs generate helix-destabilizing DNA adducts, e.g., solar UV-induced cyclobutane pyrimidine dimers (CPDs) and 6–4 pyrimidine-pyrimidone photoproducts (6-4PPs). If not efficiently removed by nucleotide excision repair (NER), these adducts block the progression of advancing replicative DNA polymerases. This, in turn, creates a state of “DNA replication stress” that precludes timely completion of S phase with potential genotoxic and carcinogenic consequences [1]. In order to alleviate these outcomes, i.e., to promote DNA synthesis restart, cells can enlist any among multiple DNA damage tolerance pathways to bypass replication-blocking lesions, including (i) error-free homologous recombination-dependent template switching [2], or (ii) error-prone translesion synthesis (TLS) following recruitment of specialized DNA polymerases to stalled replication forks (RFs) [3]. In addition, Rad51-dependent RF reversal can promote reannealing of nascent DNA [4,5]. This brings replication-blocking lesions back into double-stranded DNA, thereby providing an opportunity to repair the lesion prior to eventual resumption of normal DNA replication. In addition, recent evidence demonstrates that repriming beyond damaged bases can be used to allow continuation of DNA RF progression [6].

Following genotoxin exposure, single-stranded DNA (ssDNA) generated at stalled RF is avidly bound by heterotrimeric Replication Protein A (RPA) complexes [7]. This not only protects the ssDNA from degradation, but such RPA-bound ssDNA (hereafter RPA-ssDNA) also signals rapid activation of ataxia telangiectasia–mutated (ATM) and Rad3-related (ATR) kinase, the master regulator of intra-S phase checkpoint signaling [8,9]. ATR phosphorylates a multitude of substrates that cooperate to mitigate DNA replication stress by (i) forestalling excessive accumulation of ssDNA at, and stabilizing, stalled RF [1,10] and (ii) preventing further RF blockage by repressing the activation of new origins of replication [7,11]. In addition, RPA is recruited to all active replication origins and advancing RF in the absence of genotoxic insult, where it coats/protects ssDNA resulting from normal minichromosome maintenance (MCM) helicase activity [12]. In view of the above, maintaining an adequate supply of RPA during S phase, irrespective of whether or not cells are exposed to DNA damaging agents, is essential for timely completion of DNA synthesis [13]. Lack of ATR activity leading to unrestrained origin firing causes abnormally elevated formation of RPA-ssDNA, which, in turn, engenders progressive exhaustion of the available nuclear pool of RPA and eventual formation of lethal DSB at RF in a phenomenon termed “replication catastrophe” [14]. Moreover, as RPA is also strictly required for NER [15], conditions that promote inordinate sequestration of RPA at stalled RF and/or at aberrantly activated replication origins post-UV were shown by our lab and others to cause S phase–specific defects in the removal of UV-induced DNA photoproducts [16–19].

Several mechanisms have been shown to generate ssDNA in response to replicative stress and DNA damage: (1) During S phase, blockage of DNA polymerases causes their uncoupling from the MCM replicative helicase, which continues to unwind DNA ahead of the stalled RF, resulting in abnormally large tracts of ssDNA [20]. (2) Formation of reversed RF (5) creates nascent DNA ends that can be substrates for degradation by nucleases, e.g., MRE11 and EXO1, thereby generating ssDNA [21]. Unchecked nascent DNA degradation, termed “replication fork protection defect,” is prevented by several replicative stress response factors, including Rad51 and BRCA1/2 [22]. (3) Defects in RF reversal or lesion bypass, e.g., TLS, can increase usage of PRIMPOL-dependent repriming downstream of the lesion, which generates ssDNA “gaps” behind RFs (6). (4) Following UV exposure, excision of lesion-containing oligonucleotides during NER transiently generates short stretches of ssDNA, which can be extended by the EXO1 nuclease to promote ATR activation [23].

Given the demonstrated importance of adequate RPA availability in preventing the collapse of stalled RF [13,14], mechanisms that limit ssDNA accumulation during exposure to replication-blocking genotoxins are expected to be major determinants of genomic stability. Here, we identify genetic networks governing RPA recruitment to DNA after UV irradiation using genome-wide CRISPR-Cas9 screening. Our data highlight a heretofore unknown role for SCAI, a factor previously implicated in gene transcription and DSB repair, in modulating the cellular response to UV-induced replication stress.

## Results

### A genome-wide screen identifies regulators of RPA accumulation on DNA in response to UV irradiation

**Fig 1:**
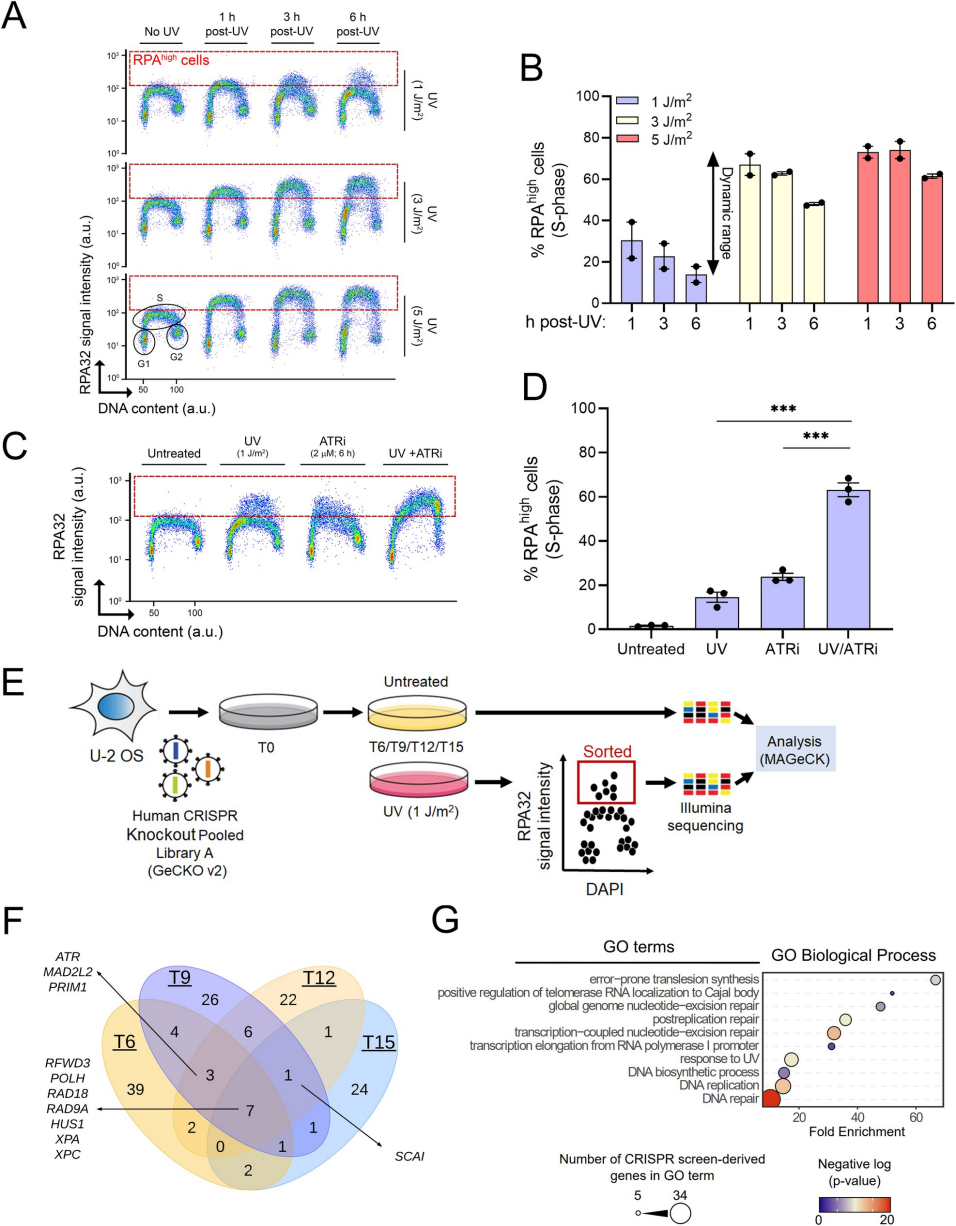
A flow cytometry–based CRISPR screen to identify regulators of RPA-bound ssDNA formation. **(A)** Immunofluorescence flow cytometry was used to measure ssDNA-bound RPA32 (y-axis) and total DNA content (x-axis; DAPI signal). Cells were treated with 1, 3, or 5 J/m^2^ UV or mock-treated, and samples were collected 1, 3, or 6 h post-UV. The dashed red box delineates DNA-bound RPA^high^ cells. (**B)** Quantification from (A). Values are the mean ± SEM from 2 independent experiments. (**C)** Proof-of-concept using the ATR inhibitor VE-821. Cells were mock-treated or irradiated with 1 J/m^2^ UV +/− 2 μM of VE-821. Samples were harvested 6 h posttreatment. (**D)** Quantification from (C). Values are mean ± SEM from 3 experiments. (**E)** Schematic overview of the FACS-based CRISPR-Cas9 screen. Cells were irradiated with 1 J/m^2^ UV at 6, 9, 12, and 15 days postinfection with the GeCKOv2 lentiviral library (see Materials and methods). At each time point, mock-treated cells were collected to assess sgRNA representation. (**F)** Venn diagram of the distribution of the genes recovered at each time point. (**G)** GO term enrichment analysis of genes identified at all time points. Statistics used: unpaired *t* test corrected for multiple comparisons using the Holm–Šídák method. **: *p* ≤ 0.01, ***: *p* ≤ 0.001. The data underlying the graphs shown in the figure can be found in S1 Data. a.u., arbitrary units ATR, Ataxia telangiectasia and Rad3-related; GO, Gene Ontology; RPA, Replication Protein A; ssDNA, single-stranded DNA.

We sought to identify gene networks that restrict RPA accumulation on DNA during genotoxin-induced replicative stress. To this end, we optimized an existing method coupling flow cytometry, stringent washes, and immunofluorescence to measure ssDNA-associated (as opposed to free) RPA32 (one of the 3 subunits of the RPA complex) in U-2 OS human osteosarcoma cells in response to 254 nm UV (hereafter UV; Fig 1A) [24]. Exposure to 1 J/m^2^ UV caused detectable RPA recruitment to DNA at 1 and 3 h post-UV, which was largely resolved by 6 h (Fig 1A and 1B). In contrast, higher UV doses (3 or 5 J/m^2^) led to persistent accumulation of RPA (close to signal saturation) at all time points post-UV that we tested (Fig 1A and 1B). The dynamic range of this assay, within a 6-h window, is therefore much larger at low (1 J/m^2^) vs higher doses of UV in U-2 OS cells (Fig 1B). As proof of principle for our experimental conditions, we treated cells with VE-821, a pharmacological ATR inhibitor that derepresses replication origins post-UV, thereby generating abundant ssDNA [13]. As expected, ATR inhibition caused a strong increase in DNA-associated RPA in response to 1 J/m^2^ UV (Fig 1C and 1D).

We devised a CRISPR-Cas9 screening strategy employing the genome-wide GeCKOv2 lentiviral library [25,26] in conjunction with the above-described flow cytometry assay (Fig 1E). U-2 OS cells were infected with the GeCKOv2 library and propagated for periods of 6, 9, 12, or 15 d to allow phenotypic expression. Cells were then either exposed to 1 J/m^2^ UV or mock-treated. At 6 h post-UV, cells were fixed and labeled with anti-RPA32 antibodies followed by FACS to sort RPA^high^ cells (i.e., within the red dotted rectangle in Fig 1A and 1C). Following extraction of DNA from untreated and RPA^high^ cells, barcode sequences were amplified by PCR, and corresponding guide RNAs (sgRNA) identified by high-throughput sequencing. Results were then analyzed using the MAGeCK pipeline to identify sgRNA that are overrepresented in the RPA^high^ population versus untreated controls [27,28].

We found that sgRNA associated with the RPA^high^ fraction changed from day 6 to 15 (Fig 1F and S1 Table), likely reflecting loss of sgRNA targeting essential and growth-promoting genes from the cell populations. Nevertheless, several genes were recovered at more than one time point (Fig 1F). Seven genes recovered at every time point encode factors with previously documented roles in the response to replicative stress and/or UV-induced DNA damage, as follows: RFWD3, a ubiquitin ligase that regulates both TLS and RPA recruitment to stalled RFs [29,30]; DNA polymerase eta, a TLS polymerase that mediates bypass of UV-induced CPD (3); RAD18, a PCNA ubiquitin ligase involved in DNA damage tolerance [31]; RAD9, a component of the intra-S phase checkpoint 911 complex [32]; and the NER pathway proteins XPA and XPC [33]. Gene Ontology (GO) term analysis of genes identified in our screen returned terms related to known pathways influencing the cellular response to UV-induced replicative stress, including error-prone TLS, nucleotide excision repair, DNA replication, and postreplication repair (Fig 1G).

**Fig 2:**
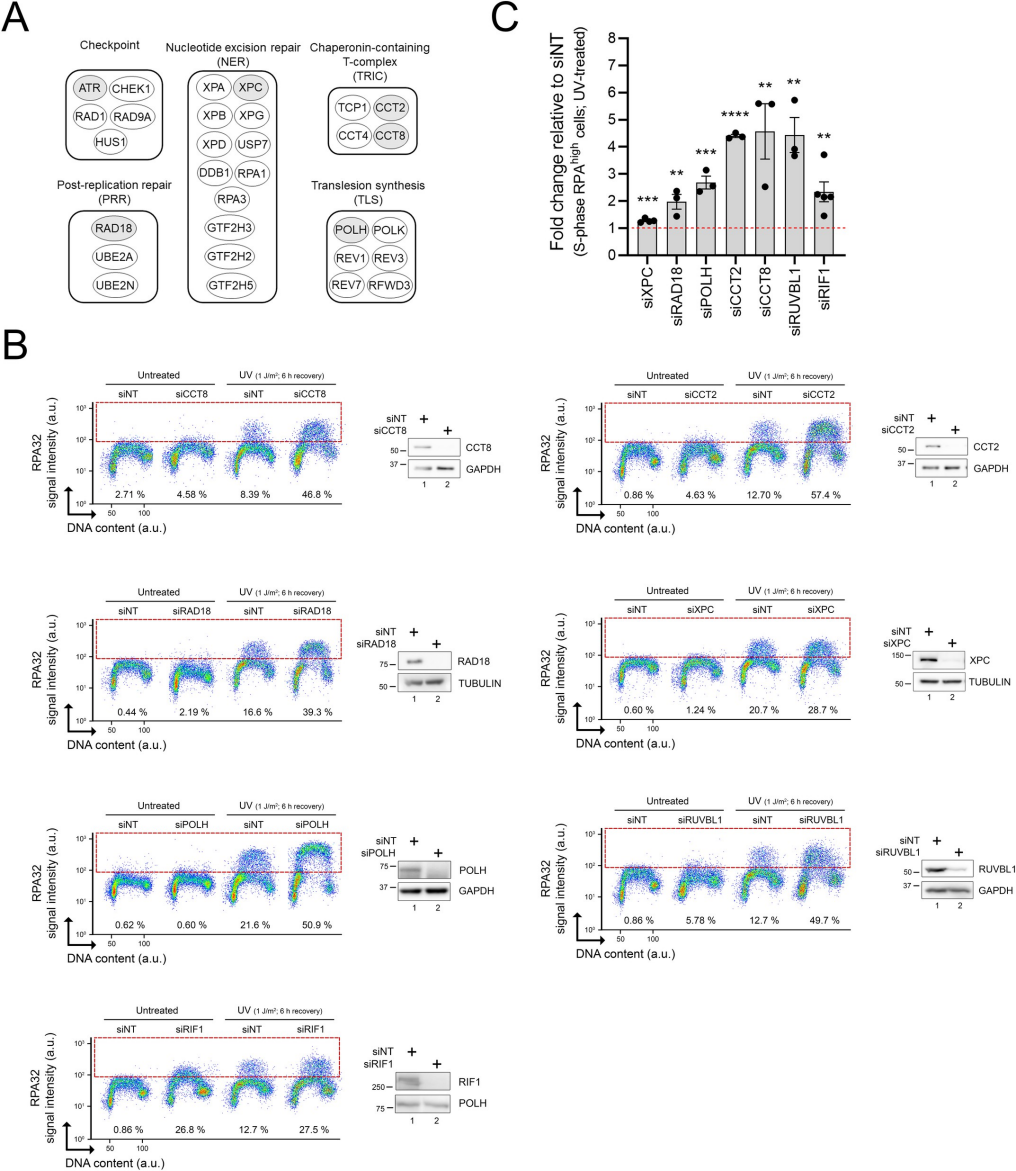
Validation of selected genes identified in the CRISPR-Cas9 screen. **(A)** Main functional groups derived from genes recovered in the screen. Genes selected for further validation are shaded in grey. (**B)** Representative immunofluorescence flow cytometry assays and immunoblots after siRNA-mediated depletion of selected genes. Cells transfected with nontargeting (siNT) or gene-specific siRNAs were mock- or UV-treated (1 J/m^2^). % RPA^high^ cells (dashed box) were assessed 6 h after irradiation. (**C)** Quantification of (B). Values represent the mean ± SEM of at least 3 independent experiments. Statistics used: unpaired *t* test corrected for multiple comparisons using the Holm–Šídák method. *: *p* ≤ 0.05, **: *p* ≤ 0.01, ***: *p* ≤ 0.001, ****: *p* ≤ 0.0001. The data underlying the graph shown in the figure can be found in S1 Data. a.u., arbitrary units; RPA, Replication Protein A.

We next evaluated siRNA-mediated depletion of individual “hits” from our screen on ssDNA-RPA formation post-UV. Genes from various functional groups were selected (Fig 2A). As expected, knockdown (KD) of RAD18, POLH, and XPC caused elevated ssDNA-RPA post-UV (Fig 2B and 2C). Our screen also identified factors whose potential roles in the UV-induced replicative stress response are incompletely characterized (Fig 2B and 2C): (i) the TRiC chaperonin complex (CCT2 and CCT8 subunits), which possesses several DNA repair/replication proteins as substrates [34]; (ii) the RUVBL1 chromatin remodeler, recently suggested to play roles in modulating the replicative stress response [35]; and (iii) RIF1, a DNA double-strand break (DSB) repair factor that also regulates DNA replication origin activity [36,37]. Down-regulation of the above factors caused elevation in RPA-ssDNA specifically in S phase cells, consistent with the notion that most of the genes recovered in our screen act by mitigating replicative stress. We note that siRNA against RIF1 also caused elevated RPA-ssDNA in the absence of UV, which might reflect the role of this gene in negatively regulating the activation of DNA replication origins in unperturbed cells [36].

Interestingly, all 3 subunits of the RPA complex were also identified as “hits” in the screen (S1 Table). This is consistent with published data indicating that partial siRNA-mediated depletion of RPA, i.e., to levels that do not compromise unchallenged DNA replication, exacerbates replicative stress [13]. As such, although it might seem counterintuitive, reduction of RPA availability was shown to ultimately elevate DNA-bound RPA in cells treated with hydroxyurea and ATR inhibitor [13]. We speculate that progressive CRISPR-Cas9-mediated depletion of RPA subunits might lead to a similar situation upon UV irradiation. In other words, while the total level of RPA is decreased due to CRISPR-Cas9 gene inactivation, the proportion of RPA that is DNA-bound is likely to be elevated due to replicative stress post-UV. Taken together, the results indicate that our screening strategy is competent in identifying mediators of the UV-induced DNA replication stress response.

### SCAI is a novel regulator of the replicative stress response

**Fig 3:**
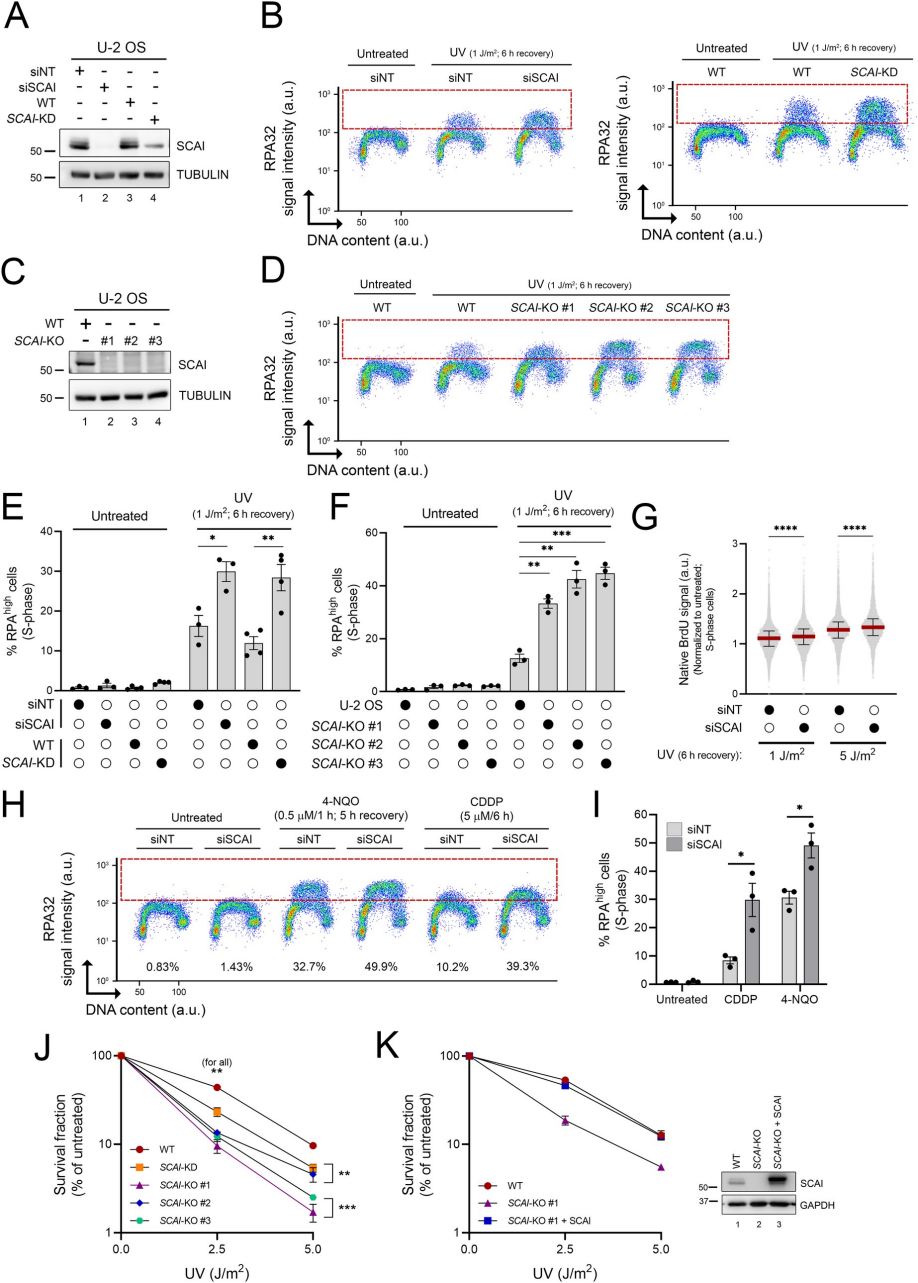
SCAI influences the replication stress response post-UV. **(A, C)** Immunoblot of whole-cell extracts from control U-2 OS, SCAI-depleted cells (A) and SCAI KO cells (C). (**B, D)** Immunofluorescence flow cytometry measurements of DNA-associated RPA32 in control, SCAI-depleted cells (B, left plot: siSCAI, right plot: SCAI-KD), or SCAI-KO (D) cells 6 h after 1 J/m^2^ UV irradiation. RPA^high^ cells are delineated by a dashed box. (**E, F)** Quantification from (B) and (D), respectively. Values are the mean ± SEM from at least 3 independent experiments. **(G)** Depletion of SCAI increases ssDNA generation post-UV. Control and SCAI-depleted cells were exposed to BrdU for 48 h, and then irradiated with UV as indicated. Native BrdU signal was assessed by immunofluorescence flow cytometry at 6 h post-UV. Median is presented (red line), and error bars indicate the interquartile range. Data combined from 2 independent experiments with similar results. (**H)** Immunofluorescence flow cytometry measurements of DNA-associated RPA32 in control and SCAI-depleted cells (as in (B)). U-2 OS transfected with siNT or siSCAI were treated with 0.5 μM 4-NQO for 1 h and allowed to recover for 5 h or continuously exposed for 6 h to 5 μM cisplatin (CPPD). (**I**) Quantification from (H). Values are the mean ± SEM from 3 independent experiments. (**J)** SCAI-KD/KO cells are sensitive to UV as measured by clonogenic survival. Values are the mean ± SEM from 3 independent experiments. (**K)** Rescue of UV sensitivity of SCAI-KO cells by transient overexpression of SCAI as determined by clonogenic survival. Values are the mean ± SEM from 2 independent experiments. Right: immunoblot of whole-cell extracts from control U-2 OS (WT), SCAI KO cells, and SCAI-KO that transiently overexpress SCAI. Statistics used: two-tailed unpaired Student *t* test (E, I), unpaired *t* test corrected for multiple comparisons using the Holm–Šídák method (J, F), and Mann–Whitney (G). *: *p* ≤ 0.05, **: *p* ≤ 0.01, ***: *p* ≤ 0.001, ****: *p* ≤ 0.0001. The data underlying the graphs shown in the figure can be found in S1 Data. 4-NQO, 4-nitroquinoline 1-oxide; a.u., arbitrary units; CDDP, cisplatin KD, knockdown; KO, knockout; RPA, Replication Protein A; ssDNA, single-stranded DNA siNT, nontargeting siRNA; siSCAI, SCAI-targeting siRNA; WT, wild type.

The SCAI gene was recovered at multiple time points in our RPA-ssDNA screen (Fig 1F). SCAI has been reported to interact with 53BP1 to modulate DSB repair [38,39] and also to influence gene transcription [40]. However, any effect of SCAI on the response to genotoxin-induced replicative stress was unknown. We found that U-2 OS cells in which SCAI was either knocked down or knocked out via CRISPR-Cas9 (SCAI-KD or SCAI-KO, respectively), or down-regulated using multiple independent siRNA, exhibited elevated RPA-ssDNA post-UV as compared to control cells (Figs 3A–3D and S1A–S1C). Like other genes identified in our screen, accumulation of RPA on DNA was observed primarily during S phase in cells lacking SCAI (Figs 3B and 3D and S1B), suggesting that this factor might modulate the response to replicative stress. siRNA-mediated SCAI depletion also caused a similar phenotype in TOV-21G ovarian cancer cells (S2A and S2B Fig).

Consistent with the elevated formation of RPA-ssDNA observed in Fig 3A–3D, native immunofluorescence of incorporated BrdU, which directly reflects ssDNA accumulation, was elevated in SCAI-depleted S phase cells post-UV as compared to control cells (Fig 3G). Exposure to the replicative stress-inducing drugs cisplatin (CDDP) and 4-nitroquinoline 1-oxide (4-NQO) was also found to increase RPA-ssDNA during S phase in SCAI-depleted compared to control cells (Fig 3H and 3I). Finally, we found that SCAI-KD and SCAI-KO cells are sensitized to UV and CDDP (Figs 3J and S1E) and that reexpression of SCAI in KO cells completely rescues their sensitivity to UV (Fig 3K). Taken together, these data show that upon exposure to genotoxins that cause replicative stress, SCAI acts to alleviate (i) abnormal accumulation of RPA-ssDNA in S phase cells and (ii) loss of cell viability and/or reduced proliferation.

**Fig 4:**
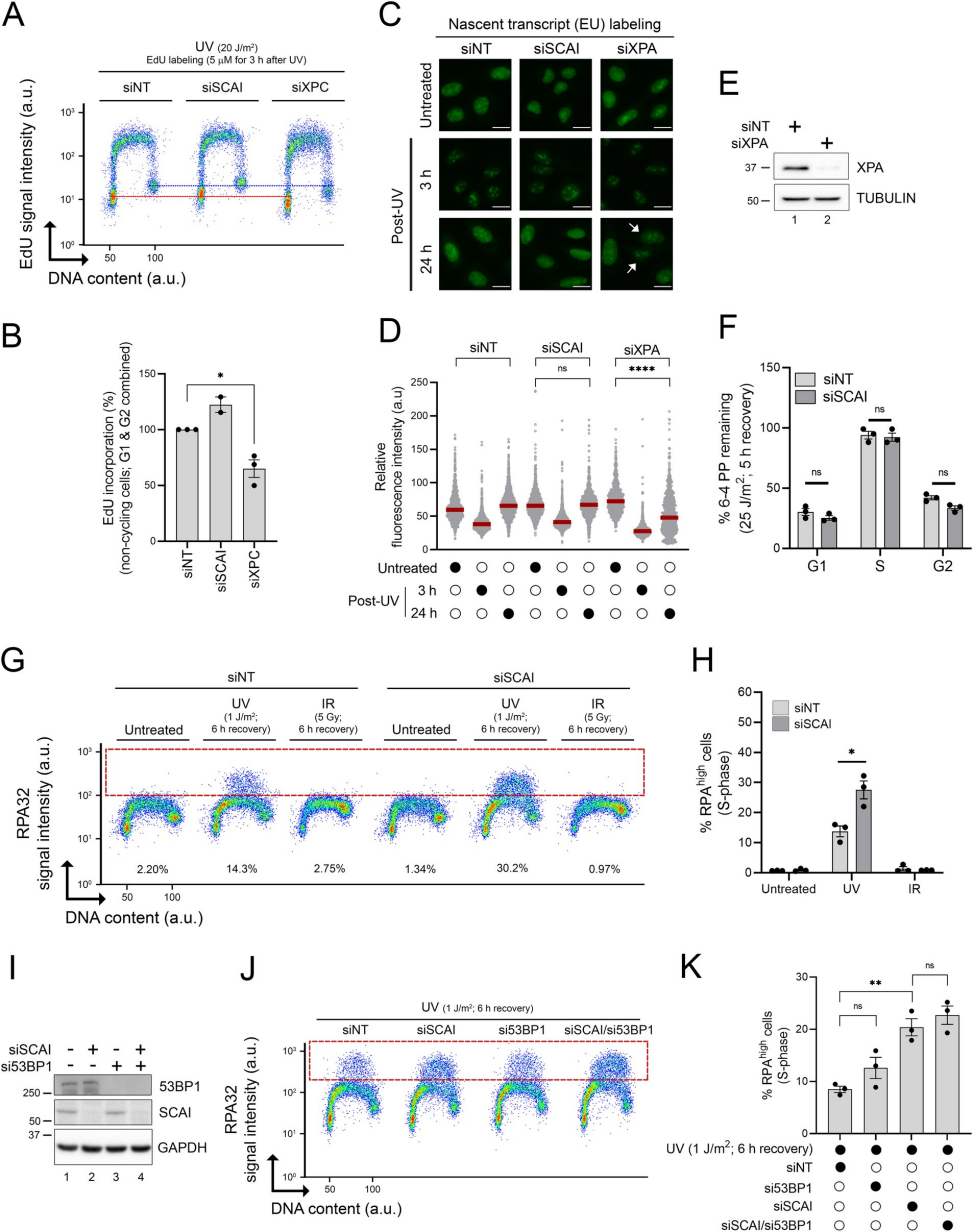
The functions of SCAI in the UV-induced replication stress response are unrelated to NER or 53BP1-dependent DSB repair. **(A)** Immunofluorescence flow cytometry was used to measure repair synthesis-associated EdU incorporation in G1/G2 cells (y-axis) and total DNA content (x-axis; DAPI signal). Cells transfected with siNT, SCAI-, or XPC-targeting siRNAs were irradiated with 20 J/m^2^ UV and allowed to recover for 3 h in medium containing 5 μM EdU. The red and blue dashed lines are positioned, respectively, in the middle of the EdU signal of the G1 and G2 cell populations of the siNT-treated cells to facilitate comparison. (**B)** Quantification from (A). Values are the mean ± SEM from at least 2 independent experiments and are relative to siNT-treated cells. (**C)** Representative images of 5-EU incorporation from cells transfected with the indicated siRNA. Cells were either mock- or UV-treated (6 J/m^2^) and samples collected 3 and 24 h after irradiation. Scale bar = 20 μM. White arrows indicate cells with reduced incorporation of 5-EU. (**D)** Quantification from (C). The red lines represent the median. The assay was repeated twice independently with similar result. (**E)** Validation of siRNA-mediated KD of XPA using immunoblot. (**F)** Quantification of UV-induced 6-4PP removal as a function of cell cycle using a flow cytometry–based assay from cells transfected with siNT or siSCAI. Values are the mean ± SEM from 3 independent experiments. (**G)** Immunofluorescence flow cytometry was used to measure DNA-bound RPA32 (y-axis) and DNA content (x-axis; DAPI signal). Cells were irradiated with either 1 J/m^2^ UV or 5 Gy IR and allowed to recover for 6 h prior to sample collection. The dashed red box delineates DNA-bound RPA^high^ cells. Representative flow cytometry plots are shown. (**H)** Quantification from (G). Values represent the mean ± SEM from 3 experiments. (**I)** Immunoblot analysis from cells transfected with the indicated siRNAs. (**J)** siNT-, siSCAI-, si53BP1-, and siSCAI/si53BP1-transfected cells were irradiated with 1 J/m^2^ UV and allowed to recover for 6 h before immunofluorescence flow cytometry analysis. The dashed red box delineates DNA-bound RPA^high^ cells. Representative flow cytometry plots are shown. (**J**) Quantification from (I). Values represent the mean ± SEM from 3 independent experiments. Statistics used: two-tailed unpaired Student *t* test (B, H), two-tailed Mann–Whitney test (D), unpaired *t* test corrected for multiple comparisons using the Holm–Šídák method (F, I). ns: nonsignificant, *: *p* ≤ 0.05, **: *p* ≤ 0.01, ****: *p* ≤ 0.0001. The data underlying the graphs shown in the figure can be found in S1 Data. a.u., arbitrary units; DAPI, 4′,6-diamidino-2-phenylindole DSB, double-strand break; EdU, 5-ethynyl-2′-deoxyuridine; EU,5-ethynyl-uridine IR, ionizing radiation; KD, knockdown; NER, nucleotide excision repair; RPA, Replication Protein A; SEM, standard error of the mean 6-4PP, 6–4 pyrimidine-pyrimidone photoproduct.

Several NER genes were recovered in our screen (Figs 1 and 2). Indeed, defective removal of UV-induced DNA lesions is expected to exacerbate RF stalling and accumulation of RPA-ssDNA in S phase cells. To address the possibility that SCAI might regulate NER efficiency, we evaluated the DNA repair synthesis step of this pathway by quantifying unscheduled incorporation of the nucleoside analog EdU in G1/G2 cells post-UV [41,42]. As expected, siRNA-mediated depletion of the essential NER factor XPC strongly attenuated repair synthesis compared to cells transfected with nontargeting siRNA (siNT) (Fig 4A and 4B). In contrast, EdU incorporation post-UV was not reduced in SCAI-depleted versus control cells, suggesting that the global genomic NER subpathway is not compromised by lack of SCAI (Fig 4A and 4B). Similarly, we found that recovery of RNA synthesis post-UV as measured by incorporation of the nucleoside analog EU [41,42], an indicator of the efficiency of the transcription-coupled NER subpathway, was similar in control versus SCAI-depleted cells but clearly defective in cells in which the essential NER factor XPA was knocked down (Fig 4C–4E). Finally, we used a flow cytometry–based NER assay, which was originally developed in our lab to directly evaluate repair of UV DNA photoproducts as a function of cell cycle [16]. We did not observe any significant difference between siNT- and SCAI-targeting siRNA (siSCAI)-treated cells with respect to removal of 6–4 photoproducts in U-2 OS cells (Figs 4F and S3). We further note that, consistent with our previously published data [16], U-2 OS cells are profoundly defective in the removal of UV-induced lesions during S phase (Fig 4F). Overall, the above results indicate that lack of SCAI does not cause replicative stress by compromising NER-mediated removal of UV-induced DNA lesions.

As mentioned previously, SCAI physically interacts with 53BP1 to modulate DSB repair [38,39]. We therefore evaluated whether this functional interaction is relevant in the context of UV-induced RPA-ssDNA accumulation in S phase cells. Compared to the situation for UV, DSB-inducing ionizing radiation (IR) did not cause noticeable accumulation of RPA on DNA in either control or SCAI-depleted cells (Fig 4G and 4H), indicating that DSB processing, i.e., end resection, does not cause significant accumulation of RPA-ssDNA in S phase cells under our experimental conditions. We further found that depletion of 53BP1, alone or in combination with that of SCAI, did not influence levels of RPA-ssDNA post-UV in our assay (Fig 4I–4K). Taken together, these data indicate that the abnormal response to replicative stress in cells lacking SCAI is unlikely to be related to defective 53BP1-dependent DSB repair.

### SCAI promotes RF progression post-UV

**Fig 5:**
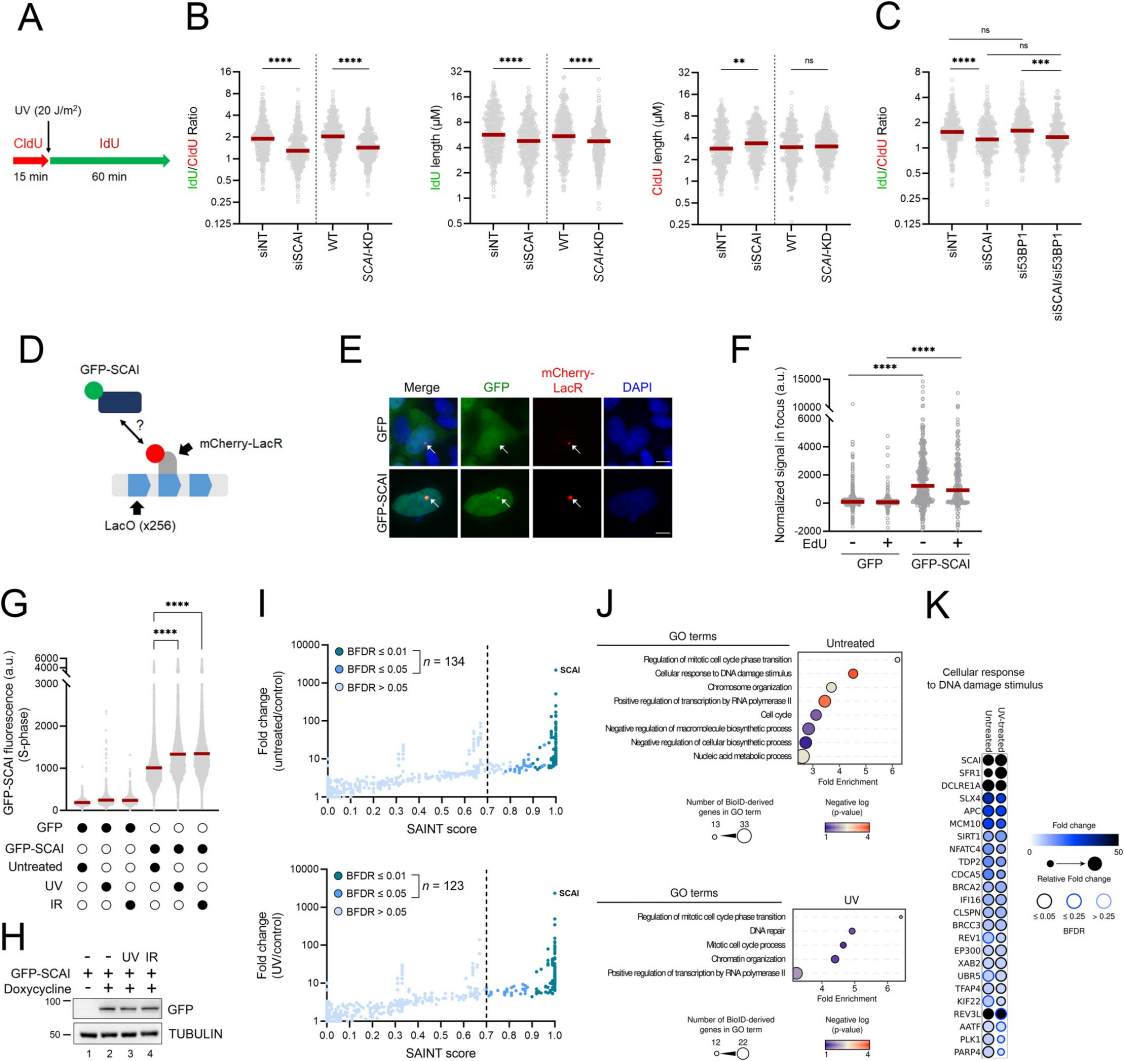
SCAI influences RF progression in cells exposed to UV. **(A)** Schematic of the DNA fiber assay used to assess RF progression post-UV. Cells were incubated with CldU (red) for 15 min, irradiated with UV (20 J/m^2^), and then incubated with IdU (green) for 60 min. (**B)** Left panel: dot plot and median (red line) of IdU/CldU ratio from control (siNT or WT) or SCAI-depleted (siSCAI or SCAI-KD) cells. Middle panel: dot plot and median of IdU tract lengths. Right panel: dot plot and median of CIdU tract lengths (data combined from *n* = 3 with similar result). (**C)** 53BP1 does not influence RF progression post-UV in cells lacking SCAI. Similar experiment as in (B) (left panel; data combined from *n* = 2 with similar result). (**D)** SCAI localizes to stalled RFs. Schematic of the assay used to evaluate recruitment of SCAI to stalled RF caused by binding of mCherry-LacR to a LacO array. (**E)** Representative microscopy images for the assay described in (D). Scale bar = 10 μM. **(F)** Quantification of GFP or GFP-SCAI normalized signal intensity in the mCherry-LacR foci in non-S phase cells (EdU−) or S phase cells (EdU+). Each point represents a single cell. Lines represent the median. Data combined from 3 similar biological replicates. (**G)** GFP-SCAI associates with DNA post-UV. Signal intensity from S phase cells was determined by flow cytometry +/− irradiation with 2 J/m^2^ UV or 5 Gy IR. Cells were allowed to recover for 6 h (UV) or 5 h (IR). Red line represents the mean. Representative results from 3 independent experiments. (**H)** Immunoblot analysis of the expression level of GFP-SCAI under the experimental conditions described in (G) ± induction by doxycycline. (**I)** Interrogation of proximity interactome was performed through biotin labeling using TurboID-SCAI under untreated and UV-treated (2 J/m^2^) conditions. Fold-change is relative to the CRAPome background controls (see Materials and methods). Proteins with a SAINT score ≥0.7 and a BFDR ≤0.05 are considered significant. **(J)** GO term enrichment analysis of proteins found in the untreated and UV-treated conditions. **(K)** Proteins associated with the GO term “Cellular response to DNA damage stimulus” are shown as a dot plot in which node color represents the fold increase, node size represents the relative fold change between the experimental conditions, and node edges represent the *SAINTexpress* BFDR. Raw data are in S2 Table. Statistics used: Mann–Whitney test (B), Kruskal–Wallis with Dunn’s multiple comparisons test (C), two-tailed unpaired Student *t* test (F), one-way ANOVA corrected for multiple comparisons using Tukey’s test (G). ns: nonsignificant, *: *p* ≤ 0.05, **: *p* ≤ 0.01, ***: *p* ≤ 0.001, ****: *p* ≤ 0.0001. The data underlying the graphs shown in the figure can be found in S1 Data. a.u., arbitrary units; BFDR, Bayesian false discovery rate; CldU, 5-chloro-2′-deoxyuridine GO, Gene Ontology; IdU, 5-iodo-2′-deoxyuridine IR, ionizing radiation; RF, replication fork; *SAINTexpress*, Significance Analysis of INTeractome; WT, wild type.

We next assessed the impact of SCAI on RF progression after UV irradiation using DNA fiber analysis. We found that both siRNA-mediated depletion and CRISPR-Cas9 KD of SCAI significantly compromised RF progression post-UV in U-2 OS cells (Fig 5A and 5B) as well as in 2 additional cancer cell lines: TOV-21G (ovarian cancer) and WM3248 (melanoma) (S2C–S2E Fig). SCAI depletion does not negatively influence RF progression in the absence of genotoxic treatment; in fact, unchallenged cells treated with siSCAI displayed modest increases in RF progression (Fig 5B). Our data also indicate that the negative impact of SCAI depletion on RF progression after UV treatment is independent of 53BP1 (Fig 5C), as was the case for SCAI-dependent modulation of RPA-ssDNA levels (Fig 4I–4K). Overall, these data demonstrate that SCAI influences RF progression after genotoxic stress.

Biochemical purification of newly replicated DNA using iPOND did not identify SCAI as a component of stalled RF [43]. Nevertheless, it remains possible that recruitment of SCAI in the vicinity of RF occurs infrequently or transiently, thereby precluding detection of SCAI using this method. We exploited a cell biology approach relying on the introduction of a 256XLacO array in U-2 OS cells expressing an mCherry-tagged LacR construct [44]. Recruitment of LacR proteins to the 256XLacOarray has been shown to be associated with induction of replicative stress markers in this genomic region [44–46]. We used cell lines harboring the 256XLacO array and expressing mCherry-LacR and either GFP-SCAI or control GFP. We confirmed that our GFP-SCAI fusion was functional by testing its ability to form nuclear foci in response to IR-induced DSB [38,39] (S4A Fig). As expected, we observed elevated signals for chromatin-bound RPA32 (reflecting ssDNA formation) and phosphorylated histone H2AX (generated by ATR and ATM kinase activity, and which can therefore reflect either replicative stress or DSB) at the LacO array in EdU-labeled (S phase) cells (S4B and S4C Fig). However, such replication stress/DNA damage markers were also observed in EdU-negative cells (S4B and S4C Fig), suggesting that replicative stress-induced DNA lesions formed at the LacO array during S may persist in subsequent phases of the cell cycle. We also observed that the intensity of GFP-SCAI colocalized with the mCherry-LacR-labeled region is significantly higher than for GFP alone in both EdU-positive and EdU-negative cells (Fig 5D–5F). Overall, the above data are consistent with the notions that (i) binding of mCherry-LacR to LacO produces localized replicative stress and (ii) that SCAI colocalizes with the LacO array in S phase cells. Since both the colocalization of SCAI with the LacO array and replication stress-induced DNA damage can be observed outside of S phase, we cannot exclude that some recruitment of GFP-SCAI to the LacO array might reflect the activity of this protein after DNA damage has been generated, e.g., during DSB repair or at postreplicative ssDNA gaps. We next tested whether SCAI is recruited to chromatin in cells experiencing UV-induced replicative stress. To this end, we used fluorescence microscopy coupled to stringent washes that remove proteins that are not bound to DNA in cells expressing either GFP alone or GFP-SCAI. We found that UV elevates the binding of SCAI to chromatin to a similar extent as IR in EdU-labeled S phase cells (Fig 5G). Importantly, our data also indicate that such significant increases in chromatin-bound SCAI post-UV do not result from elevated expression of this protein (Fig 5H). Taken together, the above data are consistent with the notion that SCAI can be recruited to genomic regions experiencing DNA replication stress.

We next sought to investigate whether SCAI can be found in close proximity to DNA repair or replicative stress proteins in vivo. To this end, we used a variation of the BioID assay (TurboID) coupled to mass spectrometry, which permits rapid biotinylation, purification, and mass spectrometry–based identification of proteins in close spatial proximity to a protein of interest (S2 Table) [47,48]. Consistent with a previous report indicating a role for SCAI in modulating transcription [40], our analysis revealed that proteins involved in gene expression and chromatin organisation are biotinylated by TurboID-SCAI both in untreated and UV-exposed cells (Fig 5I–5K and S2 Table). As expected, several peptides of the known SCAI-interacting DNA repair protein 53BP1 [38,39] were also recovered; however, the abundance of 53BP1 peptides in BioID control data sets from the Contaminant Repository for Affinity Purification database (CRAPome v2.0) [49] led to its exclusion from confirmed “hits” in our analyses. Importantly, our analyses identified several other proteins involved in DNA repair/replicative stress responses (e.g., REV1, REV3L, MCM10, BRCA2, PLK1, SLX4, UBR5, CLASPIN) as being in close physical proximity to SCAI (Fig 5K). Such proximity was observed in both UV- and mock-treated cells; while the reason for this is currently unknown, it is possible that either (i) SCAI is constitutively found in proximity/complex with the abovementioned proteins or that (ii) spontaneous DNA lesions formed in U-2 OS cells are sufficient to allow the detection of damage-induced SCAI-containing complexes. In any case, the above data support the notion that SCAI is localized in the vicinity of replicative stress response and DNA repair proteins in U-2 OS cells.

### EXO1 elevates RPA-ssDNA in the absence of SCAI

**Fig 6:**
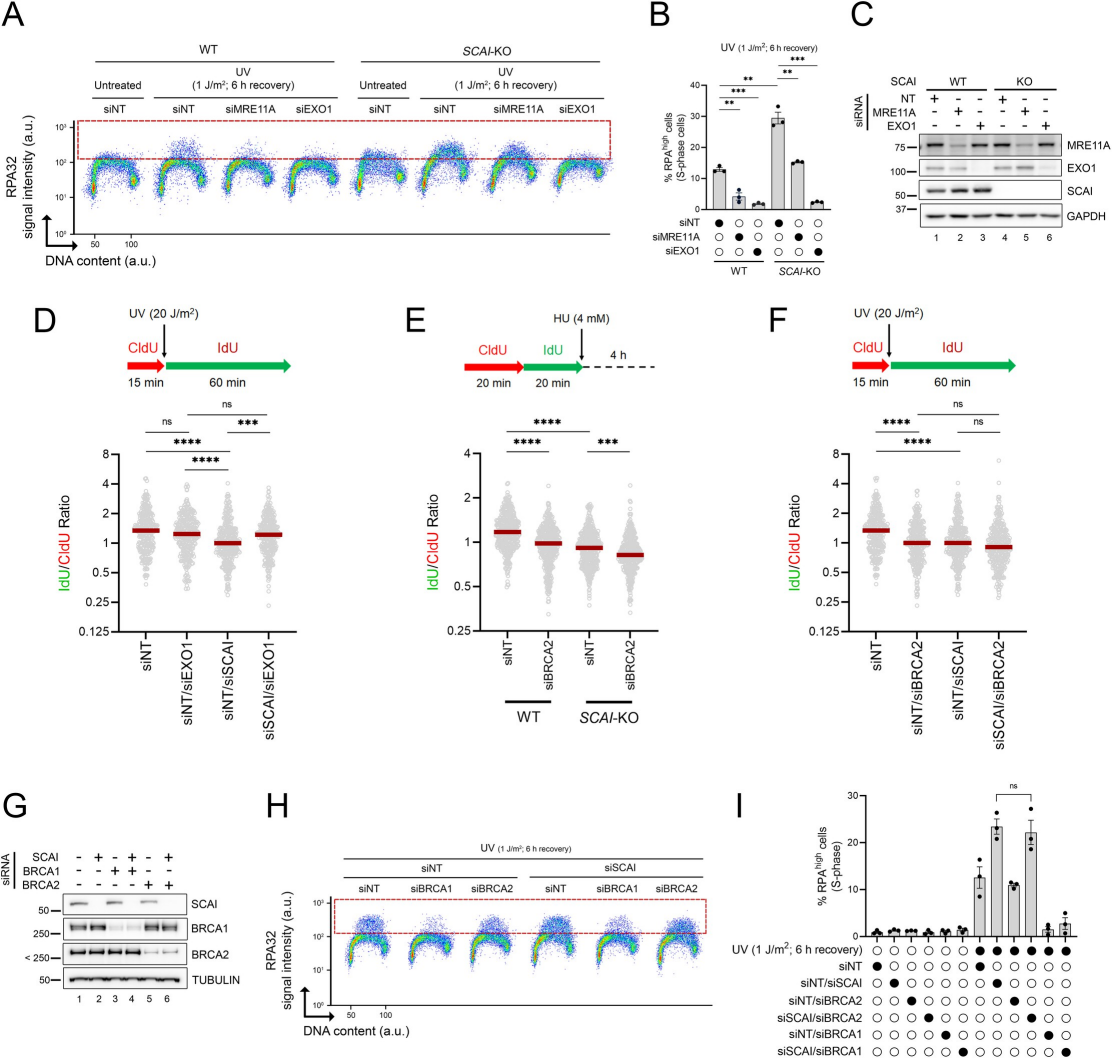
EXO1-dependent accumulation of ssDNA-RPA in cells lacking SCAI. **(A)** Depletion of EXO1, and to a lesser extent MRE11, rescues RPA-ssDNA accumulation post-UV in cells lacking SCAI. Cells were treated with 1 J/m^2^ UV or mock-treated and allowed to recover for 6 h. The dashed red box delineates DNA-bound RPA^high^ cells. (**B)** Quantification from (A). Values represent the mean ± SEM from 3 independent experiments. (**C)** Immunoblot analysis showing EXO1 or MRE11 depletions from whole-cell extracts from U-2 OS (WT) or SCAI-KO (#1) cells transfected with siRNAs. (**D)** Top: schematic of the DNA fiber assay used to assess RF progression post-UV. Cells were incubated with CldU (red) for 15 min, irradiated with UVC (20 J/m^2^), and then incubated with IdU (green) for 60 min. Bottom: dot plot of IdU/CldU ratio and median (red line) from U-2 OS transfected with siNT or siRNA against EXO1 (data combined from *n* = 2 with similar results). (**E)** Top: schematic of the DNA fiber assay to monitor RF protection defects (nascent DNA degradation) after HU. Cells were incubated successively with CldU (red) and IdU (green) for 20 min each and then exposed to 4 mM HU for 4 h. Bottom: dot plot of IdU/CldU ratio and median (red line) from U-2 OS (WT) and SCAI*-*KO (#1) cells transfected with siRNAs against BRCA2 (data combined from *n* = 2 with similar results). (**F)** Similar experiment as in (D) but from U-2 OS transfected with siNT or siRNA against BRCA2 (data combined from *n* = 2 with similar results). (**G-I)** Lack of BRCA1/2 does not cause RPA-ssDNA accumulation under our experimental conditions. **(G)** Validation of BRCA1 and BRCA2 KDs by immunoblot. (**H)** Experiments were performed as in (A) but with cells transfected with siRNAs against BRCA1 or BRCA2 +/− siSCAI. (**I**) Quantification from (H). Values are the mean ± SEM from 3 independent experiments. Statistics used: unpaired *t* test corrected for multiple comparisons using the Holm–Šídák method (B), two-tailed unpaired Student *t* test (I), Kruskal–Wallis with Dunn’s multiple comparisons test (D-F). ns: nonsignificant, **: *p* ≤ 0.01, ***: *p* ≤ 0.001, ****: *p* ≤ 0.0001. The data underlying the graphs shown in the figure can be found in S1 Data. a.u., arbitrary units; CldU, 5-chloro-2′-deoxyuridine HU, hydroxyurea; IdU, 5-iodo-2′-deoxyuridine KD, knockdown; KO, knockout; RF, replication fork; RPA, Replication Protein A; SEM, standard error of the mean ssDNA, single-stranded DNA; WT, wild type.

Several nucleases, including EXO1 and MRE11, generate ssDNA at stalled RF [22,50]. Moreover, recent reports indicate that replicative stress leads to the formation of unreplicated ssDNA gaps behind forks, which can be extended by EXO1 and MRE11 [6,51,52]. We therefore tested whether these nucleases might promote RPA-ssDNA formation in cells lacking SCAI. Strikingly, accumulation of DNA-bound RPA post-UV was abrogated upon siRNA-mediated depletion of EXO1 in SCAI-KO cells, whereas the effect of MRE11 was more modest (Figs 6A–6C and S5A). Based on this, we focused further characterization on the relationship between SCAI and EXO1-dependent DNA degradation and found that EXO1 KD rescues UV-induced RF progression defects caused by lack of SCAI (Figs 6D and S5B). We reasoned that depletion of SCAI might favor EXO1-dependent nucleolytic degradation of nascent DNA at stalled RF [50], leading to reduction in RF progression and accumulation of RPA-ssDNA. Consistent with this, we found that cells lacking SCAI displayed nascent DNA instability comparable to that caused by depletion of BRCA2 (Figs 6E and S5C) [21]. Codepletion of both factors caused an additive effect with regard to RF protection (Figs 6E and S5C), suggesting that SCAI and BRCA2 act via distinct mechanisms to protect stalled RF from nucleolytic degradation. While we observed a similar trend with respect to RF progression after UV, the additive effect caused by codepletion of BRCA2 and SCAI versus either of these proteins alone did not reach statistical significance (Figs 6F and S5D). More experiments will therefore be necessary to firmly ascertain whether these proteins act independently or redundantly in the context of UV-induced replicative stress. Taken together, the above data suggest that nascent DNA degradation is likely to contribute to the observed RF progression defects during genotoxic stress in cells lacking SCAI. Since RF protection/progression defects have been associated with sensitivity to genotoxins [53], we tested whether depletion of EXO1 might restore UV resistance in cells lacking SCAI and found that this is not the case (S5E Fig). It is therefore possible that EXO1-independent roles of SCAI, e.g., in DNA DSB repair [38,39], gene regulation [40], or heretofore unknown mechanisms, may have an overriding influence on UV sensitivity in SCAI-null cells. For example, lack of EXO1 may generate DNA lesions post-UV, e.g., DSB, which require SCAI for their processing.

We next tested directly whether RF protection defects, i.e., degradation of nascent DNA at reversed forks, contribute to the accumulation of RPA-ssDNA post-UV under our experimental conditions. As mentioned above, BRCA1/2 are well known to contribute to the protection of nascent DNA at stalled RF [21,54]. However, our screen did not identify BRCA1/2 (S1 Table), and, moreover, cells in which either of these proteins was depleted, either alone or in combination with SCAI, did not display significant elevation of RPA-ssDNA (Fig 6G–6I). Intriguingly, while depletion of BRCA2 has no significant effect on DNA-bound RPA levels post-UV, we found that lack of BRCA1 strongly suppressed ssDNA-RPA accumulation in S phase cells under our experimental conditions (Fig 6G–6I). While the basis for the latter observation is unclear, overall, our results indicate that nascent DNA degradation is unlikely to contribute to UV-induced accumulation of RPA-ssDNA in cells lacking SCAI.

**Fig 7:**
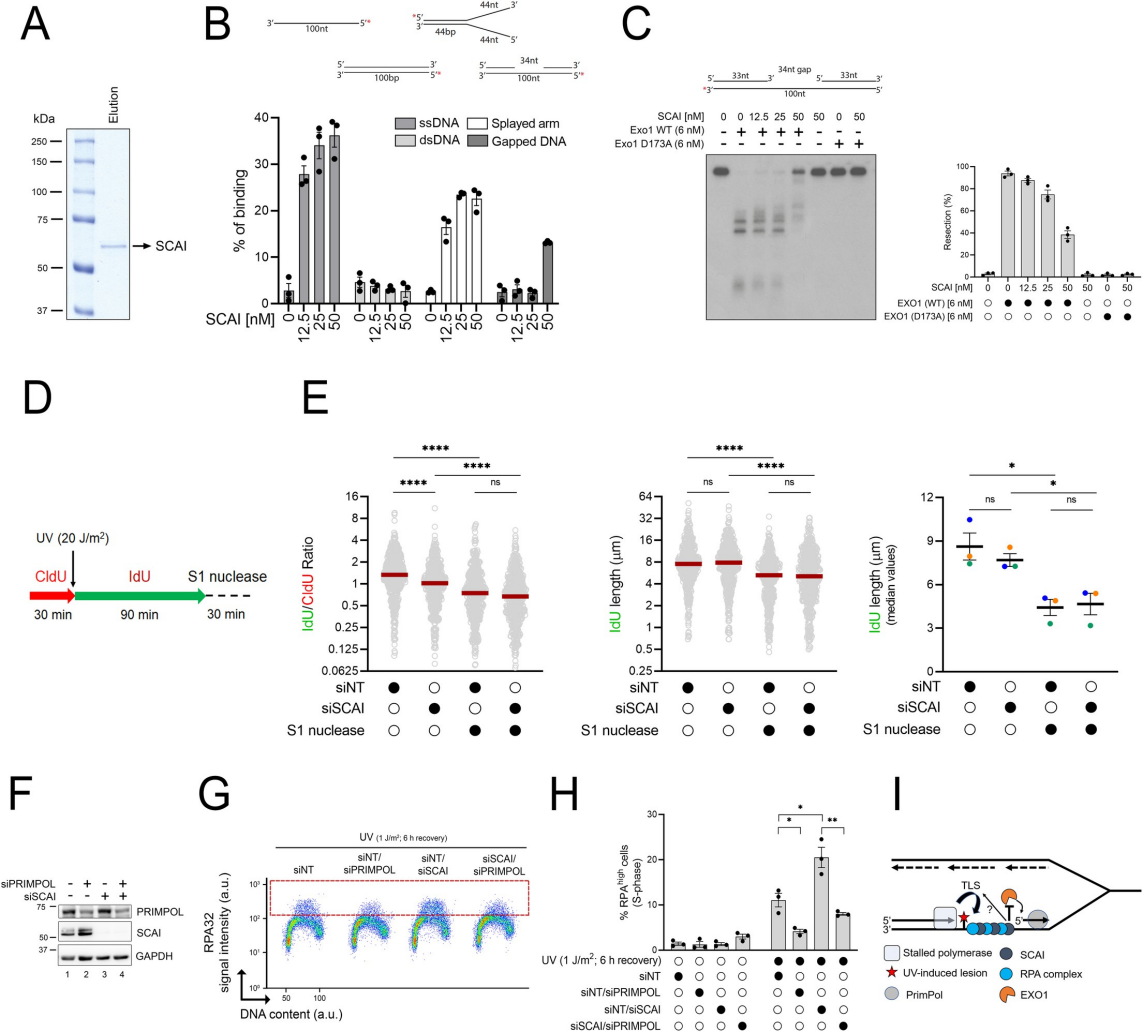
SCAI inhibits EXO1-mediated DNA resection at ssDNA gaps. **(A)** Recombinant SCAI protein was purified from insect cells, separated by SDS-PAGE and visualized by Coomassie blue staining. (**B)** SCAI preferentially binds ssDNA over dsDNA. 5′-[^32^P]-labeled ssDNA, dsDNA, splayed arm, or gapped DNA were incubated with purified recombinant SCAI at increasing concentrations and the reaction products separated by acrylamide gel electrophoresis and visualized by autoradiography (see S6A Fig). Quantification of the percentage of SCAI-mediated DNA binding on ssDNA, dsDNA, and splayed arm substrates from 3 independent experiments. (**C)** Left: in vitro DNA resection assays using a 3′-[^32^P]-labeled gapped DNA substrate in the absence of any proteins, or with WT or a catalytically inactive version of EXO1 (D173A) supplemented with purified recombinant SCAI. Right: quantification of the percentage of DNA resection from 3 independent experiments. (**D, E)** Depletion of SCAI does not increase ssDNA gap generation post-UV. (**D)** Schematic of the DNA fiber assay used to assess RF progression post-UV. Cells were incubated with CldU (red) for 30 min, irradiated with UV (20 J/m^2^), and then incubated with IdU (green) for 90 min. Cells were then treated or not with S1 nuclease. (**E)** Left panel: dot plot and median (red line) of IdU/CldU ratio from cells transfected with siRNAs as indicated. Middle panel: dot plot and median of IdU tract lengths (DNA fiber dot plot are combined from *n* = 3 with similar result). Right panel: histogram of median values of IdU track length derived from independent experiments. Means and SEM are plotted as lines and whiskers. (**F-H)** Depletion of PrimPol rescues ssDNA-RPA accumulation in cells lacking SCAI. (**F)** Validation of siRNA-mediated KD of PrimPol and SCAI by immunoblot. (**G)** Representative immunofluorescence flow cytometry plots from cells transfected with the indicated siRNA treated with 1 J/m^2^ UV or mock-treated and allowed to recover for 6 h. The dashed red box delineates DNA-bound RPA^high^ cells. (**H)** Quantification from (G). Histogram values represent the mean ± SEM from 3 independent experiments. **(I)** Proposed model. After UV exposure, PrimPol-dependent repriming generates ssDNA gaps behind RF. These gaps recruit RPA and SCAI, and SCAI acts to restrain the resection activity of EXO1. Possible modulation of Polζ/REV3L-dependent TLS by SCAI cannot be excluded. Statistics used: Kruskal–Wallis with Dunn’s multiple comparisons test (E; left and middle panels), unpaired *t* test corrected for multiple comparisons using the Holm–Šídák method (E; right panel, H). ns: nonsignificant, *: *p* ≤ 0.05, **: *p* ≤ 0.01, ****: *p* ≤ 0.0001. The data underlying the graphs shown in the figure can be found in S1 Data. a.u., arbitrary units; CldU, 5-chloro-2′-deoxyuridine; dsDNA, double-stranded DNA; IdU, 5-iodo-2′-deoxyuridine KD, knockdown; RF, replication fork; RPA, Replication Protein A; SEM, standard error of the mean; ssDNA, single-stranded DNA; TLS, translesion synthesis; WT, wild type.

EXO1 has been shown to extend ssDNA gaps left behind RF during replicative bypass of damaged DNA bases via PRIMPOL-dependent repriming [52]. Previously published data also suggest that SCAI possesses the capacity to bind ssDNA [38], raising the possibility that SCAI might directly influence the activity of EXO1 at ssDNA gaps. We purified SCAI and assessed its ability to bind various ssDNA-containing substrates in vitro (Fig 7A). Our data indicate that SCAI readily binds linear ssDNA or “splayed arm” ssDNA-containing structures but displays much lower affinity for dsDNA (Figs 7B and S6A). Interestingly, SCAI binds to a splayed arm substrate containing 44 but not 30 nt of ssDNA (Figs 7B and S6B and S6C), suggesting that the length of ssDNA influences the ability of SCAI to bind DNA. We further found that SCAI can bind a substrate containing a 34 base-long ssDNA gap (Fig 7B) and that incubation with a concentration of SCAI permitting the detection of such binding leads to significantly reduced EXO1 nucleolytic activity on this substrate (Fig 7C). In contrast, SCAI was unable to restrict EXO1 activity on the splayed arm substrate despite the ability to bind it (S6D and S6E Fig), raising the possibility that inhibition of this nuclease by SCAI requires a ssDNA/dsDNA junction, a structure present at both reversed forks and ssDNA gaps.

Lack of TLS has been shown to favor alternative pathways to tolerate DNA damage during S phase, including PRIMPOL-dependent repriming [6,51]. Interestingly, recent data indicate that SCAI interacts with the REV3L subunit of TLS polymerase zeta [55,56], raising the possibility that REV3L-dependent TLS defects might contribute to the phenotypes of SCAI by favoring the generation of ssDNA gaps post-UV upon which EXO1 can act. Concordant with the above, we found that siRNA-mediated depletion of EXO1 rescued ssDNA-RPA accumulation in cells lacking either POLH, REV1, or REV3L (S7A–S7I Fig). However, our data also indicate that siRNA-mediated depletion of both SCAI and REV3L produces additive effects on ssDNA-RPA accumulation post-UV compared to the situation for depletion of either factor alone (S7J–S7L Fig). While this result does not exclude the possibility that dysregulation of REV3L may contribute in part to the phenotypes of cells lacking SCAI, it is nonetheless compatible with the notion that SCAI does have REV3L-independent roles post-UV, e.g., modulation of EXO1-dependent ssDNA gap extension.

We next tested the impact of SCAI on ssDNA gap formation using S1 nuclease DNA fiber assays. We found that S1 nuclease-dependent reduction in the size of DNA labeled post-UV, which reflects cleavage at ssDNA gaps [57], is unchanged in SCAI-depleted versus control cells (Fig 7D and 7E), suggesting that SCAI does not influence the frequency of ssDNA gap generation. Finally, we found that siRNA-mediated depletion of PRIMPOL, which promotes repriming and postreplicative gap formation after genotoxic stress [58], rescued ssDNA-RPA accumulation post-UV in SCAI-depleted cells (Fig 7F–7H). While the above data do not exclude that SCAI might modulate TLS post-UV, they suggest that this protein acts to limit EXO1-dependent nucleolytic extension of ssDNA gaps that are formed as a consequence of repriming downstream of replication-blocking lesions (Fig 7I).

## Discussion

We developed a genome-wide screening strategy to identify genes limiting the formation of RPA-ssDNA in response to replication-blocking UV-induced DNA lesions. RPA-ssDNA serves as a platform for recruitment/activation of the intra-S phase checkpoint kinase ATR and other effectors of the replicative stress response [59,60]. One important role of the ATR-mediated intra-S phase checkpoint is to limit the generation of RPA-ssDNA during genotoxin-induced replication stress by prohibiting origin activation. This, in turn, preserves adequate pools of RPA, thereby forestalling genome-wide induction of DSB at persistently stalled RF [13,14]. We note that the precise molecular mechanisms underlying the formation of replication-associated DSB at stalled RF under conditions of limited RPA availability remain incompletely understood. ssDNA is known to be more susceptible to spontaneous cytosine deamination than dsDNA, leading to formation of abasic sites, which may promote further RF stalling if left unrepaired [61]. Moreover, ssDNA generated in the absence of ATR, which causes exhaustion of RPA pools, was found to be susceptible to cytosine deamination by APOBEC enzymes [62]. Finally, reducing the abundance of RPA stimulates the formation of secondary structures in ssDNA, which can lead to its nucleolytic degradation [63]. The literature therefore clearly indicates that ssDNA is intrinsically less stable than dsDNA and that its generation must be tightly controlled during replicative stress.

As expected, our ssDNA-RPA screen recovered several genes, which, by virtue of their participation in the activation of the intra-S phase checkpoint, are important determinants of RPA-ssDNA generation. Indeed, this signalling cascade is known to limit the accumulation of RPA-ssDNA during replicative stress in several ways. As mentioned earlier, the intra-S phase checkpoint signalling inhibits the initiation of new origins of replication, thereby restricting the number of stalled RF and consequent ssDNA formation [11,64]. Data from yeast also clearly demonstrate that intra-S phase checkpoint mutants accumulate much longer stretches of ssDNA than wild-type cells at individual stalled RF, although the precise mechanisms are not entirely clear [10]. Importantly, these stretches of ssDNA result at least in part from EXO1-dependent degradation, which is inhibited by the intra-S phase checkpoint kinase Rad53 in yeast [65].

A second category of “hits” from our RPA-ssDNA screen is involved in DNA damage tolerance via TLS. TLS polymerase eta is required for bypass of UV-induced CPD, and we previously demonstrated that lack of this enzyme causes strong accumulation of RPA on DNA post-UV [18]. Moreover, we and others showed that ssDNA accumulation is sufficiently elevated in TLS-deficient cells to cause S phase–specific defects in UV photoproduct removal by sequestering RPA away from damaged sites, thereby preventing its essential function in NER [18,19,66]. Interestingly, recently published data indicate that defective TLS enhances the formation of postreplicative ssDNA gaps by favoring PRIMPOL-dependent repriming beyond damaged bases [6,67]. Moreover, formation of such ssDNA gaps has been shown to enhance sensitivity to replicative stress [68,69]. Finally, data presented here clearly indicate that EXO1-mediated degradation, presumably at ssDNA gaps, promotes ssDNA-RPA accumulation in cells lacking TLS polymerase activity (S7 Fig). It therefore seems likely that formation of ssDNA gaps, as well as their extension via EXO1-dependent nucleolytic degradation, at least partially explains the strong representation of TLS polymerases, and regulators thereof, in our screen.

As expected, we also recovered genes encoding NER factors as determinants of RPA-ssDNA generation upon UV irradiation. In all phases of the cell cycle, NER-mediated removal of damaged DNA generates ssDNA gaps during the repair synthesis step, which can be extended via the action of nucleases [23]. Nevertheless, the absence of NER activity presumably results in a larger number of persistent replication-blocking UV-induced lesions, leading to ssDNA formation specifically in S phase cells, which is what we observed (Fig 2B). We note, however, that the extent of RPA-ssDNA generation caused by NER defects was less pronounced than those caused by deficiencies in the intra-S phase checkpoint or TLS pathways. This suggests that (i) a large fraction of persistent UV-induced DNA lesions can be readily bypassed by DNA damage tolerance pathways during S phase, and consequently, (ii) NER defects per se only cause modest elevation in replicative stress in human cells under our experimental conditions.

Our screen also identified several factors whose roles in modulating the cellular response to UV-induced replicative stress has not been as well documented compared with the above examples. TRiC is a chaperone complex that assists in protein folding [34,70,71] and has been reported to influence various cellular pathways including gene expression [72], cellular signalling [73], and protection against proteotoxic stress [74]. Interestingly, recent data indicate that activation of the integrated stress response, a signalling cascade that responds to protein misfolding, leads to inhibition of histone gene synthesis and consequent formation of R-loops that are known to inhibit DNA RF progression [75]. Curiously, however, published data also show that inhibition of RF progression caused by lack of histone synthesis is not associated with dramatic elevation of RPA-ssDNA [76]. Since TRiC assists in the folding of many proteins, the molecular mechanisms explaining its influence on DNA replication stress and RPA-ssDNA formation are likely complex, and their elucidation would require further experiments.

Rif1 plays several roles that might allow this factor to limit accumulation of RPA on DNA: (i) regulation of DSB repair by interacting with the critical nonhomologous end-joining factor 53BP1 [77]; (ii) inhibition of DNA replication origin activation by promoting dephosphorylation of the MCM complex [36,78]; and (iii) prevention of nascent DNA degradation at stalled RF [79]. Our results indicate that lack of 53BP1-dependent DSB repair does not cause an important accumulation of RPA-ssDNA in S phase cells. Furthermore, our data indicate that degradation of nascent DNA at stalled RF, i.e., defective RF protection, does not strongly contribute to RPA accumulation on DNA under our experimental conditions. We therefore speculate that, as is the case for cells lacking ATR [13], abnormal activation of DNA replication origins may contribute to elevated RPA-ssDNA generation in cells lacking Rif1. We note that our data are at odds with published reports indicating that cells lacking BRCA2, which are known to display strong fork protection defects, generate elevated ssDNA in response to hydroxyurea (HU) [80]. While the basis of this discrepancy is unknown, it is possible that degradation of nascent DNA in response to UV generates only a modest amount of ssDNA, which cannot be readily detected under our experimental conditions. We also note that our data are consistent with the demonstration that lack of BRCA1, which is well known to cause severe RF protection defects, does not elicit S phase–specific NER defects due to sequestration of RPA at stalled RF [17].

While our work was in preparation, 2 independent groups reported that SCAI interacts with the pol zeta subunit REV3L and plays important roles in the repair of DNA interstrand crosslinks [55,56]. One of these studies also found that SCAI is recruited to chromatin and promotes survival post-UV [56]. Our work is generally consistent with the aforementioned reports and, moreover, extends them by identifying SCAI as a regulator of DNA RF progression and ssDNA gap processing in response to UV-induced helix-destabilizing lesions. We note that Adeyemi and colleagues reported that lack of EXO1 modestly rescues the sensitivity of SCAI-null cells to the chemotherapeutic drug cisplatin [55]. While our results (S5E Fig) are seemingly inconsistent with this, it is possible that the impact of EXO1 on the viability of SCAI-null cells might be more important in response to drugs causing interstrand crosslinks such as cisplatin, relative to the situation for UV where these adducts are not induced. We also note that EXO1 extension of ssDNA gaps is known to promote Rad51-dependent homologous recombination and sister-chromatid exchange during replicative stress [52]. It is therefore possible that defects in such homology-dependent mechanisms in cells lacking EXO1 might ultimately cause DSB that require SCAI for their processing. Finally, our data do not exclude the possibility that compromised REV3L-dependent TLS might contribute to UV sensitivity in cells lacking SCAI, irrespective of EXO1-dependent nucleolytic extension. Indeed, REV3L-depleted cells are known to be very sensitive to UV [81].

As mentioned above, the REV3L subunit of TLS polymerase zeta was recently reported to physically associate with SCAI [55,56]. This was found to be independent of REV7, the other pol zeta subunit, suggesting that pol zeta per se is not involved in restricting ssDNA accumulation during replicative stress. While our screen did identify several TLS factors, including both REV3L and REV7, as negative regulators of ssDNA accumulation, epistasis experiments suggest that SCAI and REV3L play nonredundant roles in response to UV-induced replicative stress. Indeed, our in vitro data indicate that SCAI can act alone to limit EXO1 activity at ssDNA gaps, consistent with the notion that REV3L and SCAI exert distinct roles during ssDNA gap processing. Nevertheless, our results do not exclude the possibility that defective REV3L-dependent TLS might contribute to ssDNA-RPA accumulation in cells lacking SCAI. Further investigations will be required to evaluate the precise function of the SCAI-REV3L interaction in response to UV irradiation.

Consistent with the aforementioned recent study [55], we found that lack of SCAI leads to degradation of nascent DNA, i.e., RF protection defects. Moreover, depletion of EXO1 rescued UV-induced reduction in RF progression in cells lacking SCAI, suggesting that SCAI promotes DNA replication by limiting nucleolytic degradation of nascent DNA at stalled RF. We also note that depletion of BRCA2 in SCAI-null cells caused additive defects in RF protection upon HU treatment, suggesting that these proteins act in a nonredundant manner to protect RF from nucleolytic activity. Since nascent DNA degradation at stalled RF in cells lacking BRCA2 does not cause significant accumulation of RPA-ssDNA post-UV under our experimental conditions, RF protection defects are unlikely to account for the observed ssDNA accumulation in SCAI-KO cells. Interestingly, our in vitro data indicate that SCAI binds ssDNA with much greater affinity than dsDNA. This is in agreement with published data showing interaction of SCAI with ssDNA and with its colocalization with RPA in the context of DSB repair [38]. Moreover, we found that SCAI inhibits EXO1 activity on a ssDNA gap in vitro. Extension of ssDNA gaps by EXO1 and other nucleases has been shown to occur in response to lesions in template DNA [52] and to significantly contribute to the formation of ssDNA upon replicative stress [67–69]. Taken together, the above leads us to propose that interaction between SCAI and ssDNA at postreplicative gaps might prevent nucleolytic extension of these gaps by EXO1. While we did not formally investigate the impact of SCAI on EXO1-mediated degradation of nascent DNA at reversed forks, we note that this nuclease is known to act on both stalled RF and ssDNA gaps [50,52]. It is therefore tempting to speculate that SCAI-dependent reduction of ssDNA formation at gaps or reversed RF might entail a similar mechanistic basis. Nevertheless, further experiments will be necessary to fully characterize the mechanisms through which SCAI impacts the generation of ssDNA in human cells.

## Materials and methods

### Cell culture

U-2 OS and 293FT cells, purchased from ATCC and Invitrogen, respectively, were cultured in Dulbecco’s Modified Eagle Medium (DMEM; Gibco/Thermo Fisher) supplemented with 10% fetal bovine serum (FBS; Wisent), 2 mM L-Glutamine (Gibco/Thermo Fisher), and antibiotics (100 U/mL penicillin and 100 μg/mL streptomycin; Gibco/Thermo Fisher). U-2 OS–FokI cells (also known as U-2 OS-265), obtained from Roger Greenberg (University of Pennsylvania) [44], were cultured as above. U-2 OS Flp-In/T-Rex cells (hereafter U-2 OS FT) were cultured as above except for the addition of 100 μg/mL zeocin (InvivoGene) and 5 μg/mL blasticidin S (Gibco/Thermo Fisher) to the growth medium. Stable U-2 OS FT cell lines were maintained in the presence of 200 μg/mL hygromycin B (Gibco/Thermo Fisher) and 5 μg/mL blasticidin S. The ovarian cancer cell line TOV-21G [82] was cultured in OSE medium (Wisent) supplemented with 10% FBS and antibiotics. The WM3248 human melanoma cell line (Coriel Institute) was propagated in Eagle’s MEM (Corning) containing 15% FBS, essential and nonessential amino acids (Corning), vitamins (Corning), L-glutamine, and antibiotics. All cell lines were cultured at 37°C under 5% CO_2_ in a humidified atmosphere. Cell lines were routinely tested for mycoplasma contamination by DAPI staining/fluorescence microscopy. All cell lines were authenticated by STR analysis (McGill University Genome Center).

### Genotoxic treatment

The following drugs were used in this study: ATRi: VE-821 (Selleckchem), cisplatin (CDDP) (LKT Laboratories), HU (BioShop), 4-NQO (Sigma). Treatment conditions are indicated in the corresponding figures. For 254-nm UV exposure, cell monolayers were washed with PBS, followed by irradiation in PBS with a Philips G25T8 germicidal lamp. The fluence was 0.2 J/m^2^/s, as monitored with a DCR-100X radiometer equipped with a DIX-254 sensor (Spectroline). Cells were exposed to IR using a ^137^Cs source (Gamma Cell 3000 Elan; Atomic Energy Canada) at a dose rate of 4.5 × 10^−2^ Gy/s.

### Flow cytometry and cell sorting

Proteins bound to DNA were monitored by flow cytometry essentially as described [24]. Briefly, cells were harvested, washed once with PBS, and extracted in PBS-T buffer for 10 min on ice (0.2% Triton X-100 in PBS) to remove non-DNA-bound protein. Extracted cells were washed with PBS-B (PBS 1× + 1% BSA) and fixed in 2% formaldehyde for 30 min at room temperature. Cells were pelleted, washed with and resuspended in Perm/Wash buffer (BD Biosciences), and counted. Equal numbers of cells for each experimental condition were incubated with primary antibody (1/100) in Perm/Wash buffer for 1 h at room temperature followed by incubation with Alexa Fluor–conjugated secondary antibody (1/200) in Perm/Wash for 30 min in the dark. Click-iT chemistry was then performed to identify S phase cells, which had been labelled by adding 10 μM EdU to the cell culture medium 30 min before harvesting. Finally, cells were stained with DAPI and analyzed using an LSRII flow cytometer (BD Biosciences). The data were analyzed with FlowJo software (Flowjo LLC). Gates to assess enrichment of DNA-bound protein were established in untreated samples and applied to all samples.

### CRISPR screen

The human GEnome-scale CRISPR Knock-Out pooled library A (GeCKO v2) [25] was cotransfected into 293FT cells with the lentiviral packaging plasmids pMD2.G and psPAX2 (Addgene # 12259 and 12260, respectively). Viral production was accomplished as described previously [83] with minor modifications. Briefly, 293FT cells were cultured in complete DMEM medium without antibiotics and seeded in T-225 flasks to achieve 80% to 90% confluence at the time of transfection 1 d later. A volume of 70 μl of PLUS reagent (Invitrogen) was diluted into 2.25 mL of Opti-MEM, briefly mixed, and incubated at room temperature for 5 min. Subsequently, DNAs from the following sources were added: 30.6 μg GeCKO pooled library A, 23.4 μg psPAX2, and 15.3 μg pMD2.G. Separately, 208 μl of Lipofectamine LTX was diluted in 4.5 mL of Opti-MEM and briefly mixed. The PLUS reagent/DNA and Lipofectamine LTX mixtures were then combined, gently inverted, and incubated at room temperature for 20 min. The combined mixture was carefully added to the T-225 flask. The medium was aspirated after 24 h and replaced with harvesting media (complete DMEM + 1% BSA). Viral supernatants were harvested 48 and 72 h posttransfection, combined, filtered through a 0.45-μm Stericup filter unit (Millipore), concentrated 10× using the Lenti-X concentrator reagent, aliquoted, and frozen at −80°C.

Transduction of U-2 OS cells with the sgRNA library was performed at an MOI of 0.3 to obtain a 300× coverage. After puromycin selection, cells were maintained in exponential growth throughout the course of the CRISPR screen. To account for differences in protein depletion over time, monitoring of DNA-bound RPA^high^ cells by FACS after UV irradiation was carried out at 6, 9, 12, and 15 days posttransduction. For this purpose, 165 ×10^6^ transduced/puromycin-selected cells were seeded on fifteen 15-cm dishes 24 h prior to each time point. A control (unirradiated) dish was also included to facilitate discrimination of RPA^high^ cells. Cells were subsequently processed as described in the Flow cytometry section above. Cells displaying enrichment of DNA-bound RPA (RPA^high^) were sorted using a FACSAria cell sorter (BD Biosciences).

Genomic DNA was extracted from sorted cells as described [83]. DNA was also isolated from an aliquot of 19.5 × 10^6^ cells (= 300× coverage) harvested at every experimental time point, which serve to address the sgRNA representation throughout the CRISPR screen time course. The genomic DNA concentration was measured by fluorimetry (Turner Biosystems) using the Quant-iT PicoGreen dsDNA Assay Kit (Thermo Fisher). sgRNA sequences were amplified by PCR with the NEBNext High Fidelity PCR Master mix (NEB) using barcoded primers as described previously [84], before being subjected to next-generation sequencing on an Illumina NextSeq 550 apparatus. Raw sequencing data were processed using Cutadapt to remove adaptors [85] and trimmed to isolate 20-nt sgRNA sequences. The MAGeCK algorithm [27] was used for sgRNA sequence quantitation, gene-level enrichment, and ranking. Filtering criteria were further applied to the MAGeCK gene sets. Only genes with at least 2 positive sgRNA displaying an RRA score lower than 1 × 10^−3^ and with at least 1 sgRNA displaying a read counts difference greater than 2 between representation (total) and sorted samples (with ≥50 reads in the sorted sample) were considered for further analysis. Biological processes associated with the hit lists were analyzed with PANTHER [93] using the “statistical overrepresentation test” (annotation data set “GO biological process complete”) with Bonferroni correction for multiple testing.

### Generation of CRISPR-mediated knockdown (KD)/knockout (KO) cell lines

The U-2 OS CRISPR KD cell line was generated using the All-in-One plasmid encoding dual sgRNAs and fluorescent protein-coupled Cas9^D10A^nickase (AIO-GFP; Addgene #74119) [86]. sgRNA pairs were designed using the WTSI Genome Editing online tool (http://www.sanger.ac.uk/htgt/wge/). AIO-GFP-containing sgRNA plasmids were transfected using Lipofectamine 2000 (Life Technologies/Thermo Fisher) as per manufacturer’s instructions. Two days later, transfected (EGFP-positive) cells were individually sorted by FACS into 96-well plates at a single-cell-per-well density for clonal expansion. To derive U-2OS SCAI CRISPR knockout (KO) cell lines, sgRNA sequences obtained from the Toronto KnockOut library v3 (TKOv3; https://crispr.ccbr.utoronto.ca/crisprdb/public/library/tkov3/) were first annealed and cloned into lentiCRISPR v2 (Addgene #52961) and then used for viral production as described in the CRISPR screen section above. After transduction and puromycin selection, cells were cloned into 96-well plates through FACS. Expanded clones were evaluated by immunoblotting to confirm SCAI KD/KO status. See S3 Table for sgRNA sequences.

### siRNA transfection

For siRNA-mediated KD, cells were reverse-transfected with 20 nmol of siRNA using Lipofectamine RNAiMax (Thermo Fisher) as per manufacturer’s instructions. The medium was refreshed 24 h later, and, unless otherwise stated, experiments were carried out at 72 h posttransfection. See S3 Table for a list of siRNAs used in this study.

### Plasmids

Versions of SCAI tagged with either GFP or V5-TurboID were generated by LR cloning (Gateway) using pcDNA5-FRT-TO-EGFP (provided by Anne-Claude Gingras; University of Toronto) [87] and pcDNA5-FRT-TO-V5-TurboID, respectively, as the destination vectors. An untagged version of SCAI was generated by removing the EGFP sequence from the expression plasmid pcDNA5-FRT-TO-EGFP-SCAI through site-directed mutagenesis (Q5 site-directed mutagenesis kit, NEB). All constructs were validated by DNA sequencing.

### Generation of stable inducible cell lines

U-2 OS Flp-In/T-REx cells were transfected using Lipofectamine LTX transfection reagent (Life Technologies/Thermo Fisher). Briefly, cells were seeded at 400,000 cells/well in a 6-well plate in 2 ml of complete DMEM without antibiotics. On day 1, cells were transfected with 100 ng of the pcDNA5-FRT/TO-based expression construct and 1 μg of pOG44 (Thermo Fisher) as per manufacturer’s instructions. On day 2, the transfected cells were transferred into a 10-cm dish in complete medium and, on day 3, selected by addition of blasticidin S and hygromycin B to the growth medium. The selection medium was changed every 3 d until visible colonies were observed. Colonies were then pooled, expanded, and protein expression monitored by immunoblotting following the addition of 5 μg/mL doxycycline to the growth medium for a 24-h period.

### Clonogenic survival and growth assays

Cells were initially seeded at an appropriate density and, after attachment, washed with PBS and treated with various doses of UV in PBS. Following incubation for 14 d at 37°C, surviving colonies were stained with 0.5% methylene blue in 50% methanol. Colonies were counted and the surviving fraction was determined as follows: colonies/(seeded colonies × plating efficiency). For CDDP sensitivity, 50,000 cells were seeded overnight in 6-cm dishes. CDDP was added for 2 h in serum-free medium, followed by washing with PBS. Cells were then incubated in complete media for 3 d. After staining with 0.5% methylene blue in 50% methanol, densitometry analysis was performed to assess cell growth (using Image J). For the clonogenic survival rescue experiments upon SCAI reexpression, SCAI-KO cells were transfected with a combination of pcDNA5-FRT-TO-EGFP/pcDNA5-FRT-TO-SCAI (1:10 ratio) using Lipofectamine 2000 (Life Technologies/Thermo Fisher) as per manufacturer’s instructions. Two days later, transfected (EGFP-positive) cells were sorted by FACS and used in UV clonogenic assay as described above.

### Immunoblotting

Whole-cell extracts (WCEs) were obtained by suspending cells in the lysis buffer (25 mM Tris-HCl (pH 7.5), 2% SDS). Lysed cells were heated for 5 min at 95°C before being sonicated. Protein extracts were quantified with BCA reagent (Thermo Fisher) and analyzed by SDS-PAGE. For immunoblotting, membranes were blocked in 5% milk/TBST (TBS + 0.1% Tween-20) and then incubated with primary antibody overnight at room temperature. Membranes were subsequently probed with secondary peroxidase-conjugated antibodies that had been incubated in 5% milk/TBST at room temperature for 1 h. ECL-based chemiluminescence was detected using an Azure c600 imager (Azure Biosystems). See S3 Table for a list of antibodies used in this study.

### Quantitative RT-PCR

Total RNA was harvested using TRIzol (Thermo Fisher), DNAse-treated (RQ1; Promega), and reverse-transcribed with the SuperScript III reverse-transcriptase (Invitrogen) using random hexamers (Thermo Fisher) as per manufacturer’s instructions. For quantitative RT-PCR, cDNAs were analyzed using an ABI 7500 Real-time PCR system (Applied Biosystems) using the Luna Universal qPCR Master Mix (NEB). Results were normalized to HPRT. The 2^–ΔΔCt^ method was used to derive change in gene expression.

### DNA fiber assay

DNA fiber assays were performed essentially as described [57]. Briefly, cells were sequentially labeled with 2 thymidine analogs, 30 μM 5-chloro-2′-deoxyuridine (CldU; Sigma-Aldrich) and 250 μM 5-iodo-2′-deoxyuridine (IdU; Sigma-Aldrich) for the indicated times. Labeled cells were loaded onto glass slides and lysed in spreading buffer (50 mM EDTA, 0.5% SDS and 200 mM Tris-HCl (pH 7.4)). DNA fiber tracks were obtained through DNA spreading and fixed in 3:1 methanol:acetic acid solution for 10 min. DNA fibers were then denatured in 2.5 M HCl for 80 min, blocked for 20 min in PBS containing 5% BSA at room temperature, and sequentially stained with primary antibodies against CldU (1:400, Abcam) and IdU (1:25, BD Biosciences) for 2 h. This was followed by incubation with the corresponding secondary antibodies conjugated to various Alexa Fluor dyes for 1 h at room temperature. Lastly, slides were mounted with Immuno-Fluore (MP Biomedicals) and nascent DNA fibers visualized using a DeltaVision Elite microscope. Fiber length was measured manually using ImageJ. At least 100 DNA fibers were counted per sample. Median values are shown (red line) in all figures.

### DNA fibers with S1 nuclease treatment

DNA fiber assays with ssDNA-specific S1 nuclease were performed as described in the DNA fiber assay section with minor modifications. Cells were labeled with 30 μM CldU for 30 min, irradiated with UV, and then labeled again with 250 μM IdU for 90 min. Cells were then permeabilized with CSK100 buffer (100 mM NaCl, 10 mM MOPS (pH 7), 3 mM MgCl_2_, 300 mM sucrose, and 0.5% Triton X-100) for 10 min at room temperature, treated with the S1 nuclease (Thermo Fisher) at 20 U/mL in S1 buffer (30 mM sodium acetate (pH 4.6), 10 mM zinc acetate, 5% glycerol, 50 mM NaCl) for 30 min at 37°C, and collected by scraping in PBS-0.1% BSA. Nuclei were then pelleted at 7,000 rpm for 5 min at 4°C. The supernatant was removed leaving the volume necessary to have a final concentration of 1,500 nuclei/μl.

### SCAI foci formation post-IR

To monitor recruitment of SCAI to IR-induced foci, 400,000 U-2 OS Flp-In/T-REx cells expressing either GFP alone or GFP-SCAI were seeded on glass coverslips in a 6-well plate in doxycycline-containing media to induce protein expression. Approximately 24 h after seeding, cells were mock- or IR-treated. Cells were fixed with 4% PFA/2% sucrose for 15 min at room temperature, washed with PBS, permeabilized with CSK buffer (100 mM NaCl, 300 mM sucrose, 10 mM PIPES (pH 6.8), 3 mM MgCl_2_, 0.5% Triton-X-100), stained with DAPI, and mounted on microscopy slides for imaging. Microscopy was performed using a DeltaVision fluorescence microscope equipped with SoftWorx (GE Healthcare). Images were analyzed using ImageJ.

### Proximity labelling (TurboID)

Identification of the SCAI interaction network through spatial proteomics, using an N-terminally tagged SCAI (V5-TurboID-SCAI) construct generated by Gateway cloning from a sequence-validated entry vector, was performed essentially as described [88,89]. Briefly, polyclonal populations of stable U-2 OS Flp-In/T-REx cells with integrated V5-TurboID-SCAI were grown on 15-cm plates to 75% confluency (≈ 60 × 10^6^ cells). Bait expression was induced by addition to the growth medium of doxycycline (5 μg/mL) for 24 h. Biotinylation of potential protein partners was accomplished for 1, 3, and 6 h prior to the end of the 24-h bait expression period on UV-treated (2 J/m^2^) or mock-treated cells by the addition of 500 μM biotin to the medium. At the end of the biotinylation period, cells were kept on ice, washed extensively with cold PBS, lysed (in (50 mM Tris-HCL (pH 7.5), 150 mM NaCl, 0.4% SDS, 1% IGEPAL CA-630, 1 mM EDTA, 1.5 mM MgCl2 with Protease Inhibitors (Sigma-Aldrich #P8340) and Benzonase (Sigma-Aldrich #E8263)), sonicated, and biotinylated proteins purified with streptavidin-sepharose beads. Proteins were directly converted into peptides using the on-beads digestion technique as described [90]. Mass spectrometry analysis was performed as described [91].

### *SAINTexpress* analysis

After the removal of proteins identified as “Potential contaminant,” “Reverse,” or “Only identified by site” in the “ProteinGroups” file (from Maxquant analysis results), proteins found with at least 2 unique peptides in the sum of all experiments were selected. As negative controls, the filtered protein list was used to search into the Contaminant Repository for Affinity Purification database [49] (CRAPome, version 2.0; https://reprint-apms.org/), by selecting “*H*. *sapiens*-Proximity Dependent Biotinylation” as data set, which contains 716 control experiments. Protein-specific spectral counts from this search (minimum, average, and maximum) were used as spectral counts background controls for each filtered protein. Both the “Gene names” and “MS/MS Count” (spectral counts) from each ProteinGroups-filtered biological replicate experiment (*n* = 3) as well as CRAPome background controls were used to build the matrix necessary to run the *SAINTexpress* software (version 2.0) [92], with geometric mean of replicates as parameter to determine the Fold Change (FC) Score. *SAINTexpress* results were finally filtered by using a Bayesian false discovery rate (BFDR) cutoff of ≤0.05, a SAINT score cutoff of ≥0.70 as well as an AvgSpec ≥5 and subsequently used in ProHits-viz webtool suite (http://prohits-viz.org/) [93]. Biological processes associated with the hit lists were analyzed with PANTHER [94] using the “statistical overrepresentation test” (annotation data set “GO biological process complete”) with Bonferroni correction for multiple testing.

### Protein recruitment to LacR foci

For monitoring recruitment of GFP-tagged SCAI to mCherry–LacR–NLS foci, 150,000 U-2 OS–FokI cells were seeded on glass coverslips in a 6-well plate without induction of FokI. Approximately 24 h later, cells were transfected using 1 μg of pDEST-mCherry-LacR-NLS and 1 μg of pcDNA5-FRT-TO-(EGFP-SCAI). Approximately 48 h after transfection, cells were fixed with 4% methanol-free formaldehyde/2% sucrose for 15 min at room temperature, washed successively with PBS and CSK buffer (100 mM NaCl, 300 mM sucrose, 10 mM PIPES (pH 6.8), 3 mM MgCl_2_), and permeabilized with CSK-T buffer (CSK buffer + 0.5% Triton X-100). Cells were washed with PBS and blocked overnight (PBS, 5% BSA, 0.1% Tween-20). Fresh antibody buffer (PBS, 3% BSA, 0.1% Tween-20) containing anti-GFP, anti-RPA32, or anti-ɣ-H2A.X was subsequently added for 3 h at room temperature in a humidified chamber. Cells were rinsed 3 times with PBS followed by the addition of Alexa Fluor–conjugated secondary antibody for 1 h at room temperature in a humidified chamber. After washing with PBS, Click-iT chemistry was performed to identify S phase cells, which had been labelled by adding 10 μM EdU to the cell culture medium 30 min before the end of the experiment. Finally, cells were stained with DAPI and mounted on microscopy slides for imaging. Microscopy was performed using a DeltaVision fluorescence microscope equipped with SoftWorx (GE Healthcare). Images were analyzed using a custom Python 3.6 script. Nuclei were segmented with DAPI staining channel images using Otsu’s thresholding, followed by extraction of the average fluorescence intensity per cell in the other channels. Cells were separated into EdU(−) and EdU(+) according to their mean nuclear EdU signal. mCherry signal was thresholded using Otsu’s method to identify and segment the LacO array. Average fluorescence from other markers was then calculated within this region and compared to the average of the total nuclear value.

### Unscheduled DNA synthesis (UDS) assay

Unscheduled DNA synthesis (UDS) post-UV was monitored by flow cytometry. Briefly, cells were irradiated with UV (20 J/m^2^) and allowed to recover in complete media containing 1% FBS and 5 μM EdU for 3 h. EdU-labelled cells were then processed as described above in the Flow cytometry section. To assess the relative intensity of EdU in noncycling cells, a “dumb” negative channel was used to isolate G1 and G2 cell populations. The median value from each condition was determined and set to 100% for the siNT.

### RNA synthesis recovery (RSR) assay

Visualization of nascent transcription by 5-ethynyl-uridine (EU) labeling post-UV was performed as described [42]. Briefly, siRNA-transfected cells grown on coverslips were mock-treated or irradiated with UV (6 J/m^2^). Cells were allowed to recover for either 3 or 24 h and pulse-labelled with 400 μM of EU for 1 h prior to harvesting. In all cases, 24 h prior to the RSR assay, cells were grown in complete media containing 1% FBS to favor incorporation of EU. Labeled cells were processed as described in the above Flow cytometry section except that the concentration of Alexa647-azide was increased to 10 μM in the Click-iT reaction, and DAPI staining was performed in an analysis buffer that does not contain RNase. Microscopy was performed using a DeltaVision fluorescence microscope equipped with SoftWorx (GE Healthcare). Images were analyzed using a custom Python 3.6 script. Nuclei were segmented with DAPI staining channel images using Otsu’s thresholding, followed by extraction of the average fluorescence intensity per cell in the other channel.

### Nucleotide excision repair (NER) assay

Removal of 6-4PP as a function of cell cycle was quantified as described [16]. Briefly, siRNA-transfected cells were mock-irradiated or irradiated with 25 J/m^2^ of UV and harvested either immediately (0 h) or following a 5-h incubation period. Cells were then fixed (PBS + 2% formaldehyde + 0.2% triton X-100 + 0.3M sucrose) for 30 min at room temperature, washed in PBS-B (PBS 1× + 1% BSA), denatured with 2N HCl for 20 min at room temperature, and washed again in PBS-B. Cells were counted and an equal numbers of cells for each experimental condition were incubated with anti-6-4PP antibody in PBS-TB (PBS + 3% BSA + 0.05% Tween20) for 90 min at room temperature followed by incubation with Alexa Fluor–conjugated IgG2_a_ secondary antibody in PBS-TB for 60 min in the dark. Finally, cells were stained with DAPI and analyzed using an LSRII flow cytometer (BD Biosciences). The data were analyzed with FlowJo software (Flowjo LLC). The extent of 6-4PP removal for populations gated in each phase of the cell cycle was determined using the geometric means as follows: [(5 h) − (no UV)] / [(0 h) − (no UV)].

### Native BrdU assay

Assessment of native BrdU levels by flow cytometry was performed as described [95]. Briefly, siRNA-transfected U-2 OS cells were grown in 20 μM BrdU-containing media for 48 h before being mock- or UV-treated. Cells were then harvested, washed once with cold PBS, and fixed with cold 100% EtOH at −20°C for at least 16 h. Cells were pelleted, washed and resuspended with PBS-T (PBS 1× + 0.1% Tween-20), and counted. Equal numbers of cells for each experimental condition were blocked with PBS-F (PBS 1× + 5% FBS) for 30 min at room temperature, washed with PBS-T, and then incubated with primary antibody (anti-BrdU; 1/100) in PBS-F buffer for 2 h at room temperature followed by incubation with Alexa Fluor–conjugated secondary antibody (1/200) in PBS-F for 1 h in the dark. Finally, cells were stained with DAPI and analyzed using an LSRII flow cytometer (BD Biosciences). The data were analyzed with FlowJo software (Flowjo LLC).

### Recombinant SCAI purification

SCAI was tagged at the N-terminus with GST and at the C-terminus with His10 and was expressed and purified in Sf9 insect cells by infection with baculovirus generated from a pFASTBAC plasmid according to the manufacturer’s instructions (Bac-to-Bac, Thermo Fisher). Transfection of Sf9 cells was carried out using Cellfectin II reagent (Thermo Fisher). Sf9 cells were infected with the generated SCAI baculovirus. Approximately 72 h postinfection, cells were harvested by centrifugation and the pellet frozen on dry ice. Cells were lysed in Buffer 1 (1× PBS containing 150 mM NaCl, 1 mM EDTA, and 1 mM DTT) supplemented with 0.05% Triton X-100 and protease inhibitors. Cell lysates were incubated with 1 mM MgCl_2_ and 2.5 U/ml benzonase nuclease (Sigma-Aldrich #E8263) at 4°C for 1 h followed by centrifugation at 35,000 rpm for 1 h. Soluble cell lysates were incubated with GST-Sepharose beads at 4°C with gentle rotation. Beads were washed twice with Buffer 1 followed by incubation with Buffer 2 (Buffer 1 with 5 mM ATP, 15 mM MgCl_2_). Sepharose GST beads were washed twice with Buffer 3 (1× PBS supplemented with 200 mM NaCl) and once with P5 Buffer (20 mM NaHPO_4_, 20 mM NaH_2_PO_4_, 500 mM NaCl, 10% glycerol, 0.05% Triton-X-100, 5 mM Imidazole) followed by cleavage with PreScission protease (60 U/ml, GE Healthcare Life Sciences). The beads were applied to a column and the elution was collected and completed to 10 mL with P5 Buffer. The eluate was then incubated with TALON beads (ClonTech). Beads were washed twice with P5 Buffer and once with P30 Buffer (P5 supplemented with 25 mM Imidazole). The beads were applied to a column and the proteins eluted twice using P500 Buffer (P5 supplemented with 495 mM Imidazole). Proteins were then dialyzed in Storage Buffer (20 mM Tris-HCl (pH 7.4), 200 mM NaCl, 10% glycerol, 1 mM DTT) and stored in aliquots at −80°C.

### SCAI DNA binding assay and DNA substrates

5′-end ^32^P-labelled DNA substrates (see S3 Table for the oligonucleotide sequences of the DNA substrates) were generated using T4 PNK (NEB) and [ɣ-^32^P]ATP (PerkinElmer). End labelled JYM696 was used as the ssDNA substrate. dsDNA was produced by annealing JYM698 with labelled JYM696, the splayed arm DNA was generated by annealing labelled JYM925 with JYM926 (short arms; 30 nt) or labelled JYM5782 with JYM5783 (long arm; 44nt), while the 34 nt gapped DNA was made by annealing labelled JYM696 with JYM5735 and JYM5736.

### DNA binding assays

The DNA-binding reactions (10 μl) contained the indicated DNA substrates (100 nM) and the indicated concentrations of purified SCAI in MOPS buffer (25 mM MOPS (pH 7.0), 60 mM KCl, 0.2% Tween-20, 2 mM DTT, and 5 mM MgCl_2_). Reaction mixtures were incubated at 37°C for 15 min and transferred on ice. The reactions were subjected to electrophoresis on an 8% polyacrylamide gel at 4°C. Gels were dried for 35 min at 85°C on Whatman paper and visualized by autoradiography. Densitometric analyses were performed using a FLA-5100 phosphoimager (Fujifilm) and quantified using the Image Reader FLA-5000 v1.0 software. Briefly, for each lane, total and bound DNA band signals were quantified. Background corresponding to unoccupied areas of the gel was subtracted. Then, the amount of bound DNA was expressed as the percentage of the total lane signal.

### In vitro resection assays with SCAI and EXO1

The 3′-end ^32^P-labelled gapped DNA was generated using JYM696 and TdT (NEB), while the 5′-end ^32^P-labelled splayed arm DNA (44 nt) was made using JYM5782 and T4 PNK (NEB). In vitro reactions were conducted using the splayed arm or gapped DNA probe in standard buffer (20 mM HEPES (pH 7.5), 0.1 mM DTT, 0.05% Triton X-100, 100 μg/mL BSA) with 2 mM ATP and 5 mM MgCl_2_. Reactions were initiated on ice by adding the indicated concentrations of purified SCAI and transferred immediately to 37°C for 5 min to allow binding of SCAI on the DNA substrates. Subsequently, 6 nM purified EXO1 WT or EXO1 D173A (Exonuclease-dead) were added and transferred immediately to 37°C for 30 min. Reactions were stopped by proteinase K treatment for 30 min at 37°C. Products were analyzed on an 8% (for the gapped DNA) or a 15% (for the splayed arm DNA substrate) denaturing polyacrylamide/urea gel. Gels were dried for 2 h at 85°C on Whatman paper and visualized by autoradiography. Densitometric analyses were performed using a FLA-5100 phosphoimager (Fujifilm) and quantified using the Image Reader FLA-5000 v1.0 software. Briefly, for each lane, total and resected DNA band signals were quantified. Background corresponding to unoccupied areas of the gel was subtracted. Then, the amount of resected DNA was expressed as the percentage of the total lane signal.

### Statistics and reproducibility

Details of the individual statistical tests are indicated in the figure legends as follows: ns: nonsignificant, *: *p* ≤ 0.05, **: *p* ≤ 0.01, ***: *p* ≤ 0.001, ****: *p* ≤ 0.0001. All experiments were repeated at least twice unless otherwise noted. Statistical differences in DNA fiber ratio or tract lengths were determined either by Kruskal–Wallis followed by Dunn’s multiple comparisons test or by Mann–Whitney test. Statistical differences for the other experiments were determined by two-tailed unpaired Student *t* test or by one-way ANOVA followed by Tukey’s multiple comparison test.

## Supporting information

S1 Fig SCAI functions in the replication stress response. **(A)** KD efficiency of SCAI using 2 independent siRNAs was evaluated by immunoblotting. (**B)** Representative immunofluorescence flow cytometry plots after siRNA-mediated depletion of SCAI. Cells were mock- or UV-treated (1 J/m^2^). % RPA^high^ cells (dashed box) were assessed 6 h after irradiation. (**C)** Quantification from (B). Histogram values represent the mean ± SEM from 3 independent experiments. (**D)** Quantification of RPA32 signal intensity from cells transfected with siNT or a pool of siRNAs against SCAI from EdU− (G1 and G2) and EdU+ (S phase) cells that were UV-treated (1 J/m^2^) and allowed to recovered for 6 h. Data represent the combination of *n* = 3 similar biological replicates. Red lines represent the mean. (**E)** SCAI-KD cells are sensitive to CDDP. Cells were treated for 2 h with CDDP in serum-free medium, followed by washing with PBS. Cells were then incubated in complete media for 3 days. Densitometry analysis of images of the stained dishes was used to evaluate cell growth. Statistics used: two-tailed unpaired Student *t* test (C), one-way ANOVA corrected for multiple comparisons using Tukey’s test (D), two-tailed unpaired Student *t* test (E). ns: nonsignificant, *: *p* ≤ 0.05, **: *p* ≤ 0.01, ***: *p* ≤ 0.001 ****: *p* ≤ 0.0001. The data underlying the graphs shown in the figure can be found in S1 Data. a.u., arbitrary units; CDDP, cisplatin KD, knockdown; RPA, Replication Protein A; SEM, standard error of the mean siNT, nontargeting siRNA.
(TIF)

S2 Fig SCAI functions in the replication stress response in cellular backgrounds other than U-2 OS. **(A)** Representative immunofluorescence flow cytometry plots after siRNA-mediated depletion of SCAI in TOV-21G cells. Cells were mock- or UV-treated (6 J/m^2^). % RPA^high^ cells (dashed box) were assessed 6 h after irradiation. (**B)** Quantification from (A). (**C)** Schematic of the DNA fiber analysis. Cells were incubated with CldU (red) for 15 min, irradiated with UV (20 J/m^2^), and further incubated with IdU (green) for 60 min. (**D, E)** SCAI down-regulation caused UV-induced reduction of RF progression in TOV-21G ovarian cancer (D) and WM3248 melanoma cell lines (E). (**D, E)** Top: dot plot of IdU/CldU ratio and median (red line) from siNT and siSCAI-transfected cells (combination from *n* = 2 with similar results). Bottom: validation of siRNA-mediated KD of SCAI by immunoblot. Statistics used: Mann–Whitney test. **: *p* ≤ 0.01, ****: *p* ≤ 0.0001. The data underlying the graphs shown in the figure can be found in S1 Data. CldU, 5-chloro-2′-deoxyuridine; IdU, 5-iodo-2′-deoxyuridine; KD, knockdown; RF, replication fork; RPA, Replication Protein A siNT, nontargeting siRNA; siSCAI, SCAI-targeting siRNA.
(TIF)

S3 Fig The function of SCAI in the UV-induced replication stress response is unrelated to NER. Representative immunofluorescence plots for removal of 6-4PP by flow cytometry. U-2 OS cells transfected with siNT or siSCAI were treated with 25 J/m^2^ UV (or mock-UV) and collected immediately (0 h) or at 5 h post-UV. Cells were labeled with anti-6-4PP antibody and DAPI. Rectangles show gating of each phase of the cell cycle according to DAPI. Geometric means of 6-4PP signal in each phase were used to calculate the percentage of 6-4PP remaining at 5 h in Fig 4F. DAPI, 4′,6-diamidino-2-phenylindole NER, nucleotide excision repair; siNT, nontargeting siRNA; siSCAI, SCAI-targeting siRNA; 6-4PP, 6–4 pyrimidine-pyrimidone photoproduct.
(TIF)

S4 Fig SCAI localizes to damaged chromatin. **(A)** Functional validation of the GFP-SCAI construct as assessed by the recruitment of SCAI to IR-generated DSB repair foci. U-2 OS Flp-In/T-REx cells with a stably integrated GFP-SCAI construct were exposed to IR (5 Gy) and fixed/imaged after an incubation period of 5 h. Representative microscopy images are shown. Scale bar = 20 μM. (**B)** Top: schematic of the assay used to evaluate recruitment of GFP-SCAI, RPA32, and ɣ-H2A.X to the LacO array. Bottom: representative microscopy images. Scale bar = 20 μM. (**C)** Quantification of RPA32 or ɣ-H2A.X normalized signal intensity in the mCherry-LacR foci in non-S phase cells (EdU−) or S phase cells (EdU+). Each point represents a single cell. Lines represent the median. Data are the combination of *n* = 3 similar biological replicates. (**D)** Interrogation of proximity interactome was performed through biotin labeling using TurboID-SCAI under untreated and UV-treated (2 J/m^2^) conditions. Proteins recovered are shown as dot plots in which node color represents the fold increase, node size represents the relative fold change between the experimental conditions, and node edges represent the *SAINTexpress* BFDR. Raw data can be found in S2 Table. Statistics used: Kruskal–Wallis with Dunn’s multiple comparisons test (B). ****: *p* ≤ 0.0001. The data underlying the graphs shown in the figure can be found in S1 Data. a.u., arbitrary units; BFDR, Bayesian false discovery rate; DSB, double-strand break; IR, ionizing radiation; *SAINTexpress*, Significance Analysis of INTeractome.
(TIF)

S5 Fig Impact of SCAI on RF dynamics. **(A)** Quantification of DNA-bound RPA^high^ cells from cells treated with 1 J/m^2^ UV and collected 1, 3, or 6 h post-UV. Values are the mean ± SEM from 3 independent experiments. (**B)** Top: schematic of the DNA fiber assay used to assess RF progression post-UV. Cells were incubated with CldU (red) for 15 min, irradiated with UVC (20 J/m^2^), and then incubated with IdU (green) for 60 min. Bottom: dot plot of IdU/CldU ratio and median (red line) from U-2 OS and SCAI-KD cells transfected with siNT or siRNA against EXO1 (data combined from *n* = 2 with similar results). (**C)** Top: schematic of the DNA fiber assay to monitor RF protection defects (nascent DNA degradation) after HU. Cells were incubated successively with CldU (red) and IdU (green) for 20 min each and then exposed to 4 mM HU for 4 h. Bottom: dot plot of IdU/CldU ratio and median (red lines) from U-2 OS (WT) and SCAI*-*KD cells transfected with siRNA against BRCA2 (data combined from *n* = 2 with similar results). (**D)** Similar experiment as in (B) but from cells transfected with siNT or siRNA against BRCA2 (data combined from *n* = 2 with similar results). (**E)** UV sensitivity of SCAI-KO (#1) cells is not rescued by siRNA-mediated depletion of EXO1. Values are the mean ± SEM from 2 independent experiments. Statistics used: Kruskal–Wallis with Dunn’s multiple comparisons test (B-D). ns: nonsignificant, ***: *p* ≤ 0.001 ****: *p* ≤ 0.0001. The data underlying the graphs shown in the figure can be found in S1 Data. CldU, 5-chloro-2′-deoxyuridine; HU, hydroxyurea; IdU, 5-iodo-2′-deoxyuridine; KD, knockdown; KO, knockout; RF, replication fork; SEM, standard error of the mean; WT, wild type.
(TIF)

S6 Fig SCAI preferentially binds ssDNA over dsDNA. **(A)** 5′-[^32^P]-labeled ssDNA, dsDNA, splayed arm (44 nt arm length), or gapped DNA were incubated with purified recombinant SCAI at increasing concentrations and the reaction products separated by acrylamide gel electrophoresis and visualized using autoradiography. (**B)** Similar experiment as in (A) but using a splayed arm DNA substrate with 30 nt arm length. (**C)** Quantification of SCAI binding from (B). (**D)** In vitro DNA resection assays using the 5′-[^32^P]-labeled splayed arm DNA substrate (44 nt arm length) in the absence of any proteins, with WT or a catalytically inactive version of EXO1 (D173A) supplemented with purified recombinant SCAI. (**E)** Quantification of the percentage of DNA resection from (D). Cartoons of the various substrates are shown on top of their respective gel. Autoradiographs are representative results from 3 independent experiments. The data underlying the graphs shown in the figure can be found in S1 Data. dsDNA, double-stranded DNA ssDNA, single-stranded DNA; WT, wild type.
(TIF)

S7 Fig Influence of TLS on ssDNA-RPA accumulation in cells lacking SCAI. **(A-C)** Depletion of EXO1 rescues DNA-bound RPA formation in cells lacking Polη (A), REV1 (B), or REV3L (C) post-UV. Representative immunofluorescence flow cytometry plots used to measure RPA32 (y-axis) and total DNA content (x-axis; DAPI signal). Cells were treated with 1 J/m^2^ UV or mock-treated and samples were collected 6 h post-UV. The dashed red box delineates DNA-bound RPA^high^ cells. (**D-F)** Quantification from (A-C). (**G-I)** KD efficiency of siRNAs from (A-C) was confirmed by immunoblotting (G-H) or RT-qPCR (I). (**J-L)** Depletion of SCAI increases the level of DNA-bound RPA32 in cells lacking REV3L. Representative immunofluorescence flow cytometry plots were used to measure RPA32 (y-axis) and total DNA content (x-axis; DAPI signal). Cells were treated with 1 J/m^2^ UV or mock-treated, and samples were collected 6 h post-UV. The dashed red box delineates DNA-bound RPA^high^ cells. (**K)** Quantification from (J). (**L)** Representative quantification of RPA32 signal intensity from cells in mid and late S phase. Lines represent the mean. Values in bar graphs represent the mean ± SEM from 3 independent experiments. Statistics used: unpaired *t* test corrected for multiple comparisons using the Holm–Šídák method (D-F, I, and K), one-way ANOVA corrected for multiple comparisons using the Tukey method (L). *: *p* ≤ 0.05, **: *p* ≤ 0.01, ***: *p* ≤ 0.001, ****: *p* ≤ 0.0001. The data underlying the graphs shown in the figure can be found in S1 Data. a.u., arbitrary units; KD, knockdown; RPA, Replication Protein A; RT-qPCR, quantitative real-time PCR; SEM, standard error of the mean ssDNA, single-stranded DNA; TLS, translesion synthesis;
(TIF)

S1 Data Excel spreadsheet containing the underlying data for Figs 1B, 1D, 1G, 2C, 3E, 3F, 3G, 3I, 3J, 3K, 4B, 4D, 4F, 4H, 4I, 5B, 5C, 5F, 5G, 5I, 5J, 6B, 6D, 6E, 6F, 6I, 7B, 7C, 7E, 7H, S1C, S1D, S1E, S2B, S2D, S2E, S4C, S5A, S5B, S5C, S5D, S5E, S6C, S6E, S7D, S7E, S7F, S7I, S7K and S7L. (XLSX)

S1 Raw Images Original blot images for Figs 2B, 3A, 3C, 3K, 4E, 4I, 5H, 6C, 6G, 7C, 7F, S1A, S2D, S2E, S6A, S6B, S6D, S7G and S7H. (PDF)

S1 Table Read counts and MAGeCK-derived results for each experimental time points of the CRISPR-Cas9 screen. Related to Fig 1E–1G.
(XLSX)

S2 Table Proximity labelling mass spectrometry raw data and SAINTexpress-derived results from untreated and UV-treated samples. Related to Figs 5I–5K and S4D.
(XLSX)

S3 Table List of antibodies, siRNAs, oligonucleotides sequences, cell lines, and plasmids. (XLSX)

## Abbreviations

ATM: ataxia telangiectasia–mutated
ATR: Ataxia telangiectasia and Rad3-related
BFDR: Bayesian false discovery rate
CldU: 5-chloro-2′-deoxyuridine
CDDP: cisplatin
CPD: cyclobutane pyrimidine dimer
DMEM: Dulbecco’s Modified Eagle Medium
DSB: double-strand break
EdU: 5-ethynyl-2′-deoxyuridine
EU: 5-ethynyl-uridine
FBS: fetal bovine serum
GO: Gene Ontology
HU: hydroxyurea
IdU: 5-iodo-2′-deoxyuridine
IR: ionizing radiation
KD: knockdown
KO: knockout
MCM: minichromosome maintenance
NER: nucleotide excision repair
RF: replication fork
RPA: Replication Protein A
RSR: RNA synthesis recovery
siNT: nontargeting siRNA
siSCAI: SCAI-targeting siRNA
ssDNA: single-stranded DNA
TLS: translesion synthesis
UDS: unscheduled DNA synthesis
WCE: whole-cell extract
4-NQO: 4-nitroquinoline 1-oxide
6-4PP: 6–4 pyrimidine-pyrimidone photoproduct
