# Supplementary figures and images for: A genome-wide screen identifies SCAI as a modulator of the UV-induced replicative stress response
Source: PLoS Biol. 2022 Oct 10;20(10):e3001543. doi: 10.1371/journal.pbio.3001543 (PMC9584372; doi:10.1371/journal.pbio.3001543)

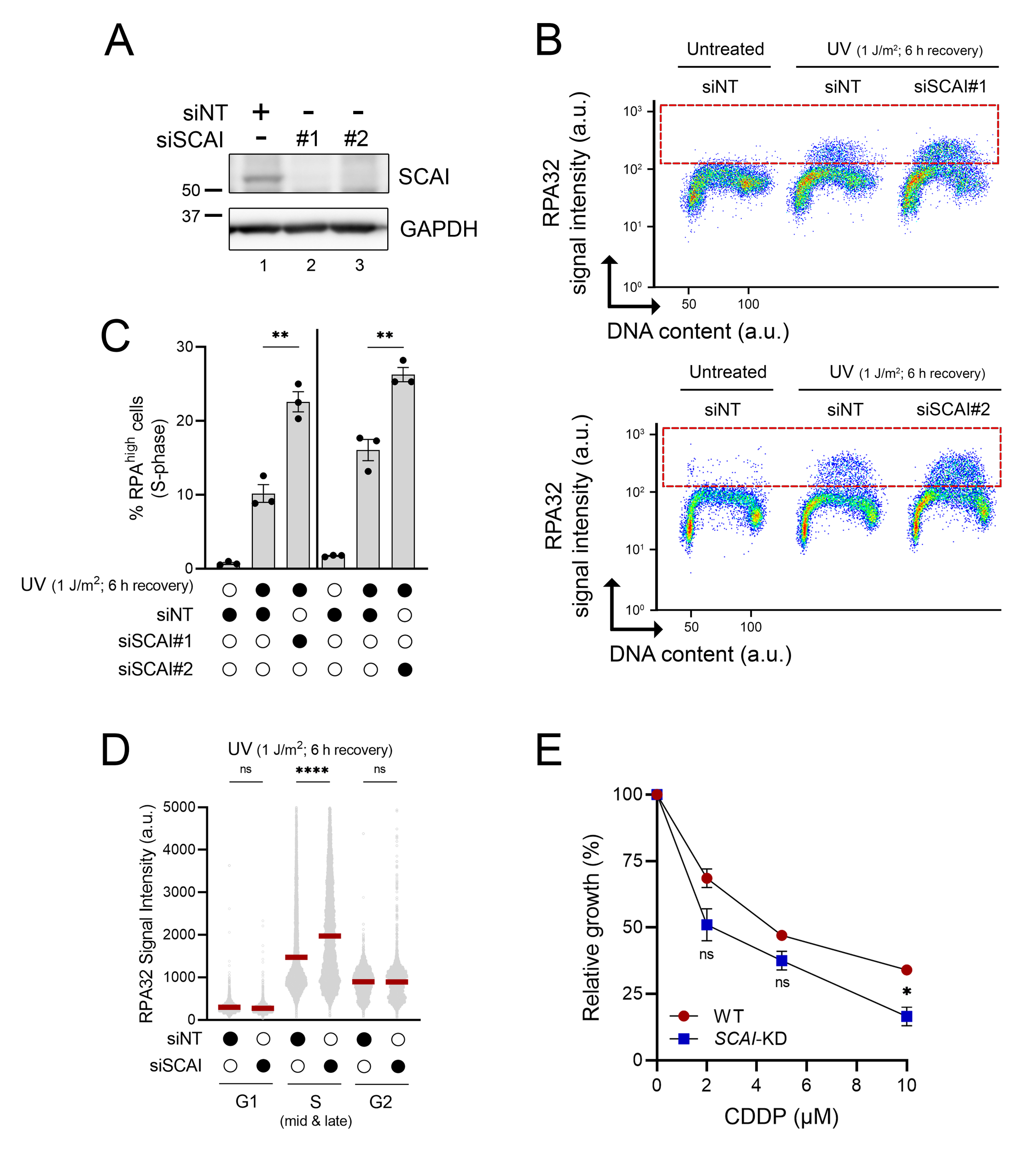

Supplement: S1 Fig — (A) KD efficiency of SCAI using 2 independent siRNAs was evaluated by immunoblotting. (B) Representative immunofluorescence flow cytometry plots after siRNA-mediated depletion of SCAI. Cells were mock- or UV-treated (1 J/m2). % RPAhigh cells (dashed box) were assessed 6 h after irradiation. (C) Quantification from (B). Histogram values represent the mean ± SEM from 3 independent experiments. (D) Quantification of RPA32 signal intensity from cells transfected with siNT or a pool of siRNAs against SCAI from EdU− (G1 and G2) and EdU+ (S phase) cells that were UV-treated (1 J/m2) and allowed to recovered for 6 h. Data represent the combination of n = 3 similar biological replicates. Red lines represent the mean. (E) SCAI-KD cells are sensitive to CDDP. Cells were treated for 2 h with CDDP in serum-free medium, followed by washing with PBS. Cells were then incubated in complete media for 3 days. Densitometry analysis of images of the stained dishes was used to evaluate cell growth. Statistics used: two-tailed unpaired Student t test (C), one-way ANOVA corrected for multiple comparisons using Tukey’s test (D), two-tailed unpaired Student t test (E). ns: nonsignificant, *: p ≤ 0.05, **: p ≤ 0.01, ***: p ≤ 0.001 ****: p ≤ 0.0001. The data underlying the graphs shown in the figure can be found in S1 Data. a.u., arbitrary units; CDDP, cisplatin KD, knockdown; RPA, Replication Protein A; SEM, standard error of the mean siNT, nontargeting siRNA. (TIF) [file pbio.3001543.s001.tif]

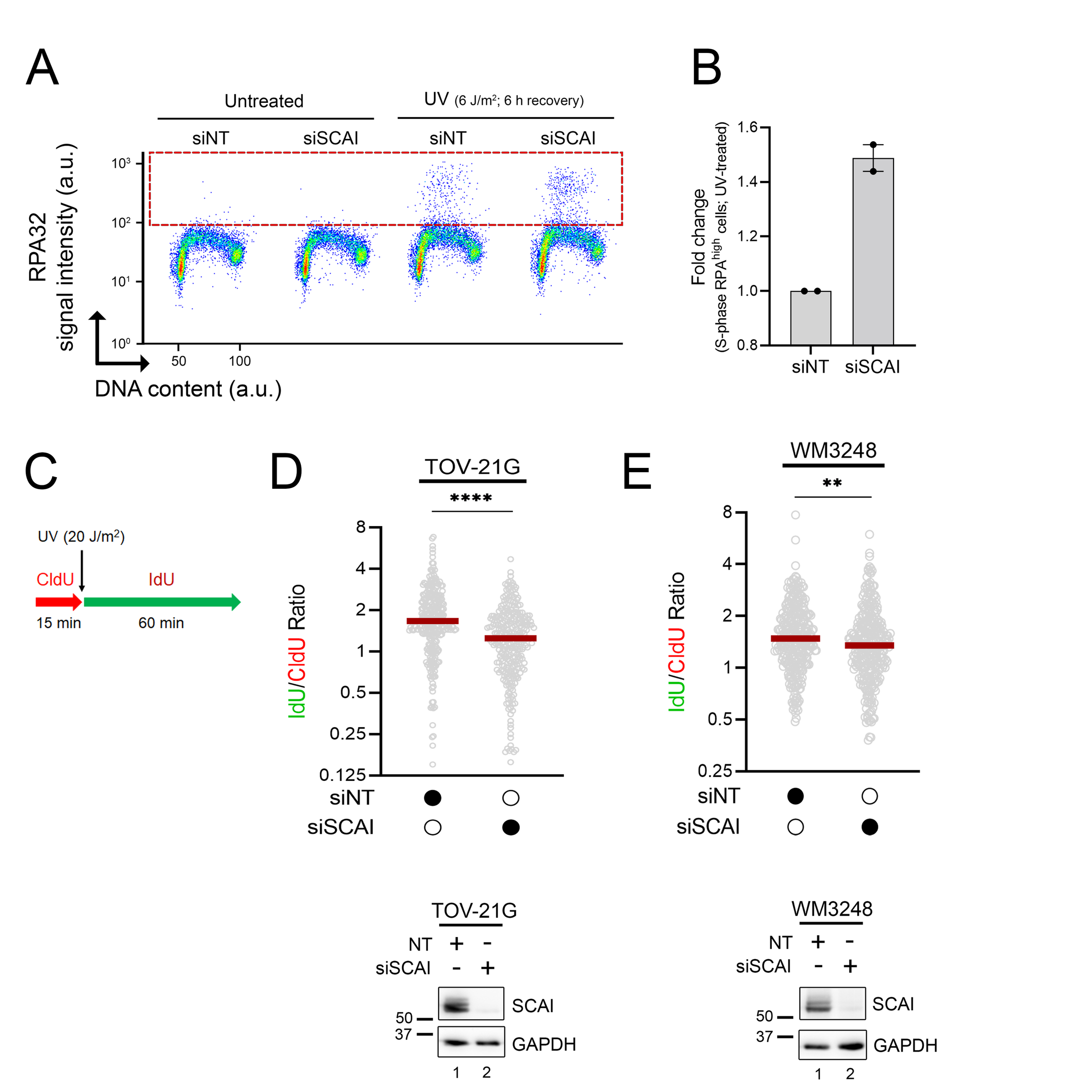

Supplement: S2 Fig — (A) Representative immunofluorescence flow cytometry plots after siRNA-mediated depletion of SCAI in TOV-21G cells. Cells were mock- or UV-treated (6 J/m2). % RPAhigh cells (dashed box) were assessed 6 h after irradiation. (B) Quantification from (A). (C) Schematic of the DNA fiber analysis. Cells were incubated with CldU (red) for 15 min, irradiated with UV (20 J/m2), and further incubated with IdU (green) for 60 min. (D, E) SCAI down-regulation caused UV-induced reduction of RF progression in TOV-21G ovarian cancer (D) and WM3248 melanoma cell lines (E). (D, E) Top: dot plot of IdU/CldU ratio and median (red line) from siNT and siSCAI-transfected cells (combination from n = 2 with similar results). Bottom: validation of siRNA-mediated KD of SCAI by immunoblot. Statistics used: Mann–Whitney test. **: p ≤ 0.01, ****: p ≤ 0.0001. The data underlying the graphs shown in the figure can be found in S1 Data. CldU, 5-chloro-2′-deoxyuridine; IdU, 5-iodo-2′-deoxyuridine; KD, knockdown; RF, replication fork; RPA, Replication Protein A siNT, nontargeting siRNA; siSCAI, SCAI-targeting siRNA. (TIF) [file pbio.3001543.s002.tif]

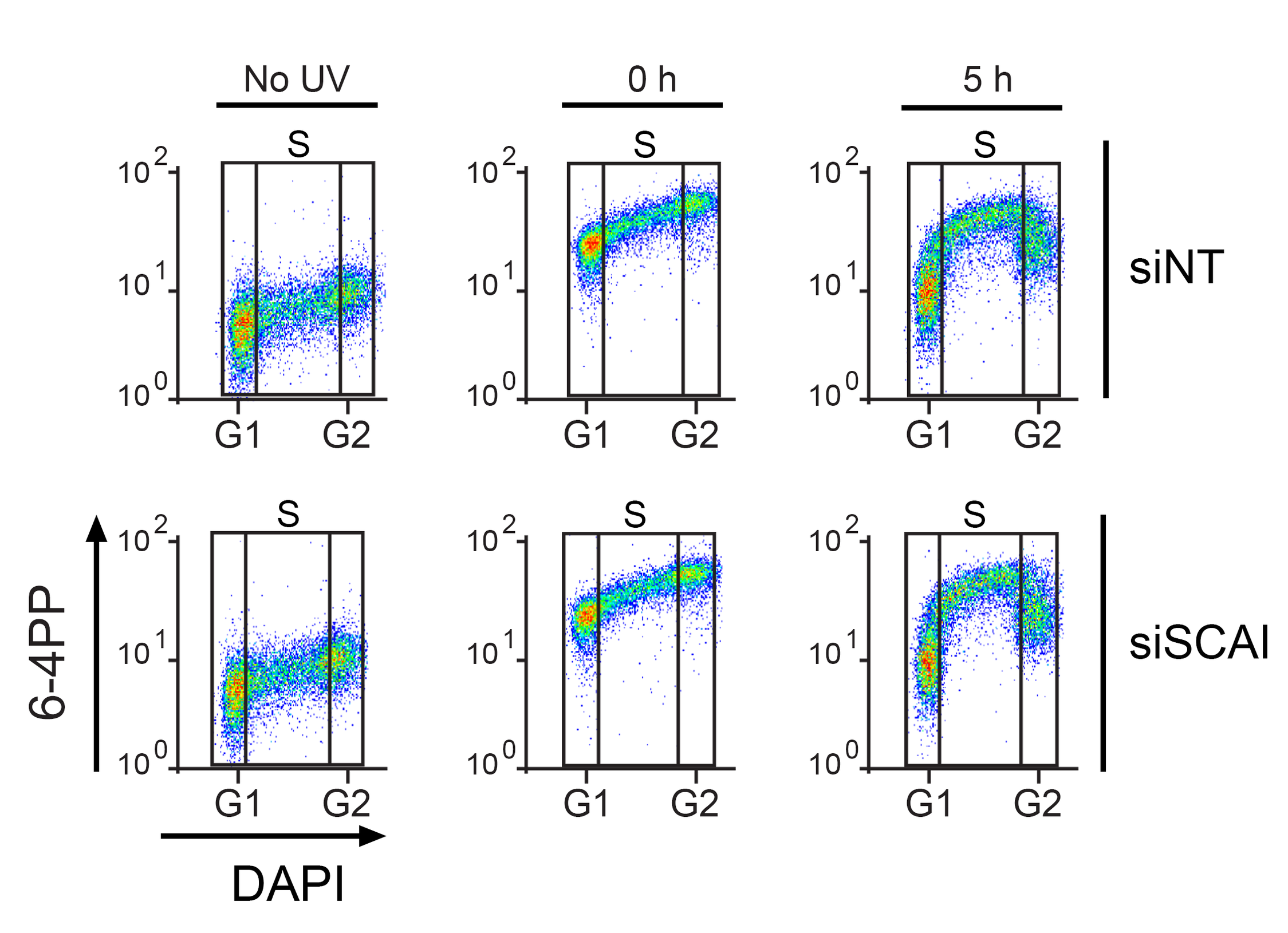

Supplement: S3 Fig — Representative immunofluorescence plots for removal of 6-4PP by flow cytometry. U-2 OS cells transfected with siNT or siSCAI were treated with 25 J/m2 UV (or mock-UV) and collected immediately (0 h) or at 5 h post-UV. Cells were labeled with anti-6-4PP antibody and DAPI. Rectangles show gating of each phase of the cell cycle according to DAPI. Geometric means of 6-4PP signal in each phase were used to calculate the percentage of 6-4PP remaining at 5 h in Fig 4F. DAPI, 4′,6-diamidino-2-phenylindole NER, nucleotide excision repair; siNT, nontargeting siRNA; siSCAI, SCAI-targeting siRNA; 6-4PP, 6–4 pyrimidine-pyrimidone photoproduct. (TIF) [file pbio.3001543.s003.tif]

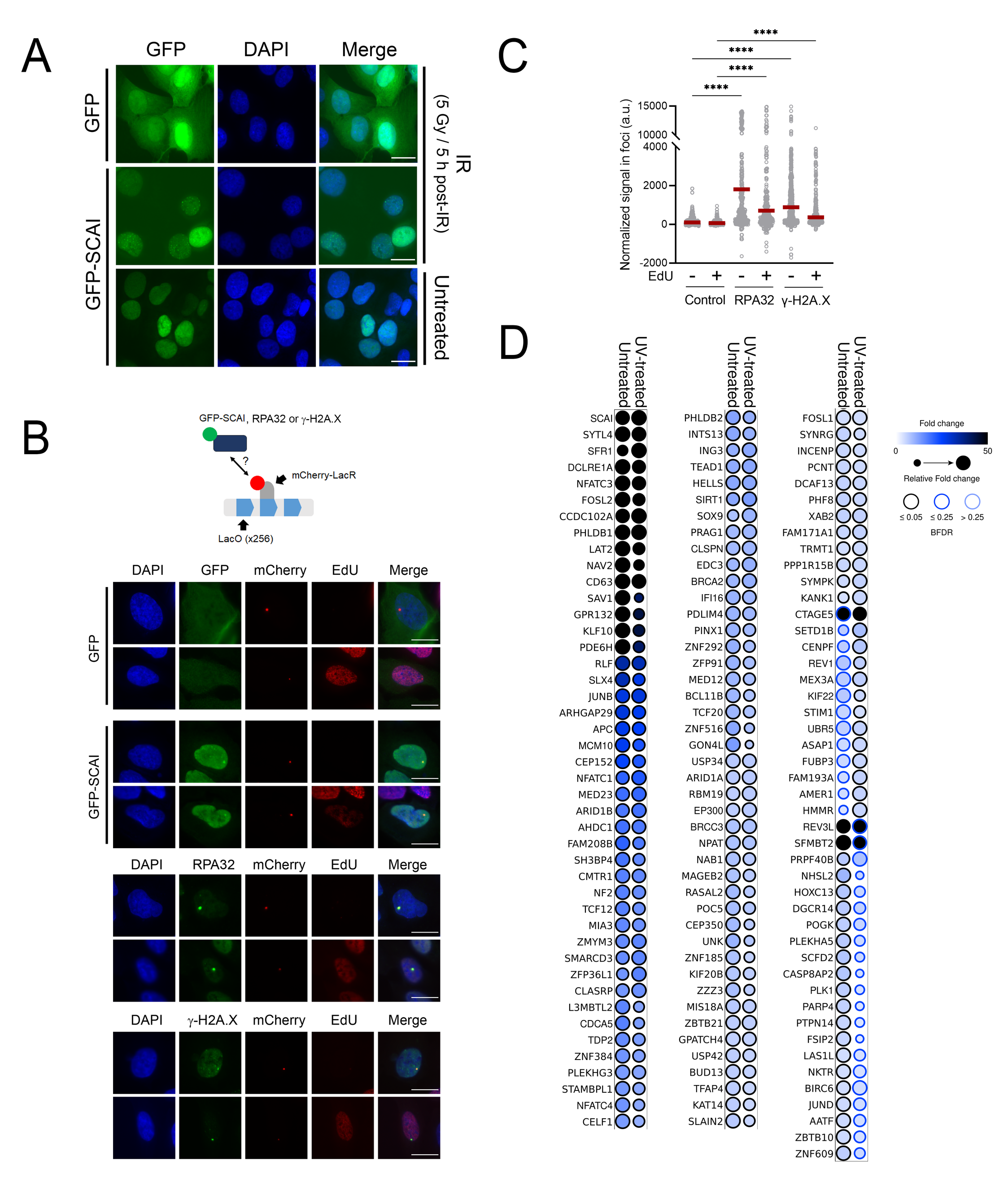

Supplement: S4 Fig — (A) Functional validation of the GFP-SCAI construct as assessed by the recruitment of SCAI to IR-generated DSB repair foci. U-2 OS Flp-In/T-REx cells with a stably integrated GFP-SCAI construct were exposed to IR (5 Gy) and fixed/imaged after an incubation period of 5 h. Representative microscopy images are shown. Scale bar = 20 μM. (B) Top: schematic of the assay used to evaluate recruitment of GFP-SCAI, RPA32, and ɣ-H2A.X to the LacO array. Bottom: representative microscopy images. Scale bar = 20 μM. (C) Quantification of RPA32 or ɣ-H2A.X normalized signal intensity in the mCherry-LacR foci in non-S phase cells (EdU−) or S phase cells (EdU+). Each point represents a single cell. Lines represent the median. Data are the combination of n = 3 similar biological replicates. (D) Interrogation of proximity interactome was performed through biotin labeling using TurboID-SCAI under untreated and UV-treated (2 J/m2) conditions. Proteins recovered are shown as dot plots in which node color represents the fold increase, node size represents the relative fold change between the experimental conditions, and node edges represent the SAINTexpress BFDR. Raw data can be found in S2 Table. Statistics used: Kruskal–Wallis with Dunn’s multiple comparisons test (B). ****: p ≤ 0.0001. The data underlying the graphs shown in the figure can be found in S1 Data. a.u., arbitrary units; BFDR, Bayesian false discovery rate; DSB, double-strand break; IR, ionizing radiation; SAINTexpress, Significance Analysis of INTeractome. (TIF) [file pbio.3001543.s004.tif]

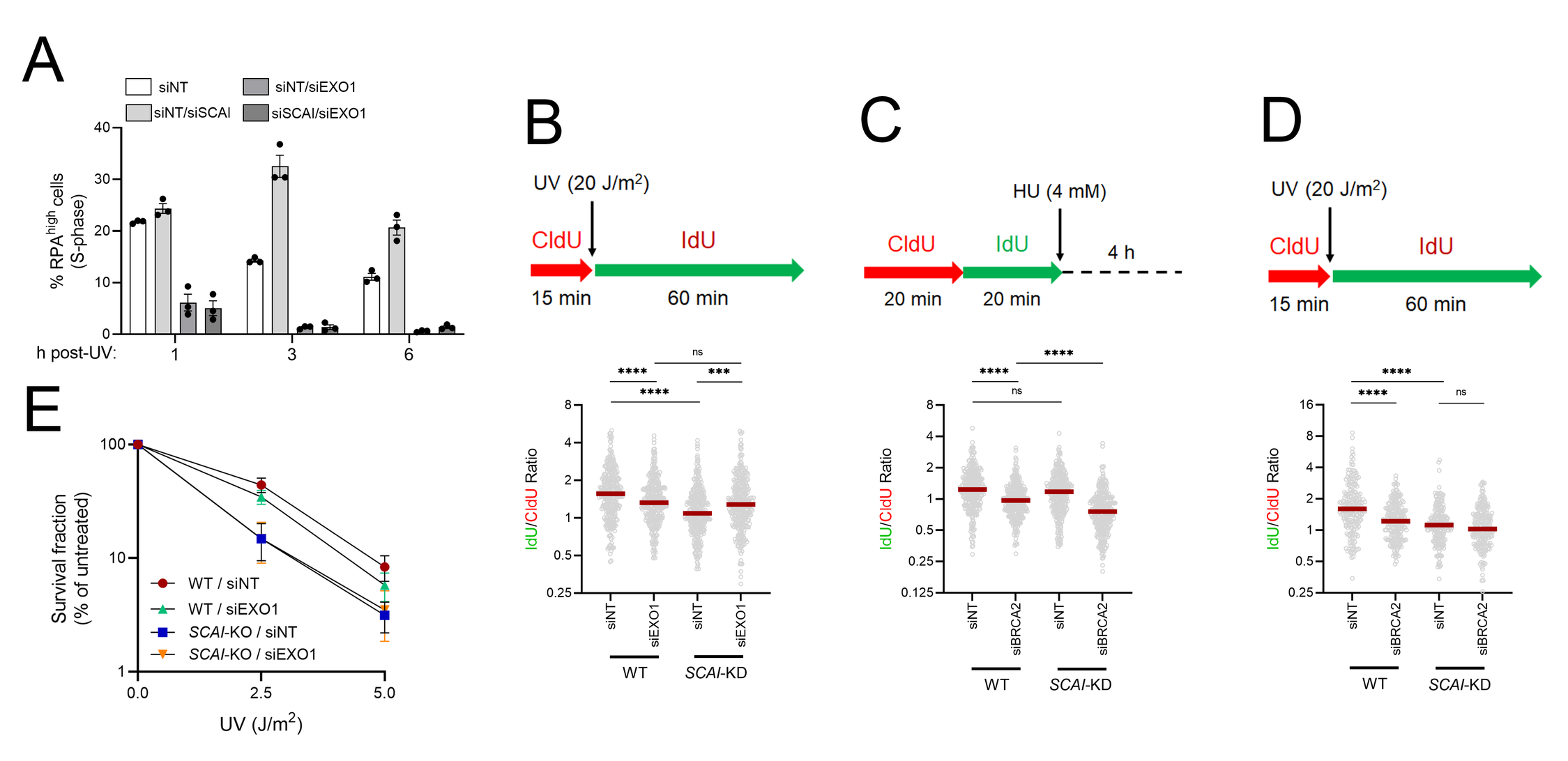

Supplement: S5 Fig — (A) Quantification of DNA-bound RPAhigh cells from cells treated with 1 J/m2 UV and collected 1, 3, or 6 h post-UV. Values are the mean ± SEM from 3 independent experiments. (B) Top: schematic of the DNA fiber assay used to assess RF progression post-UV. Cells were incubated with CldU (red) for 15 min, irradiated with UVC (20 J/m2), and then incubated with IdU (green) for 60 min. Bottom: dot plot of IdU/CldU ratio and median (red line) from U-2 OS and SCAI-KD cells transfected with siNT or siRNA against EXO1 (data combined from n = 2 with similar results). (C) Top: schematic of the DNA fiber assay to monitor RF protection defects (nascent DNA degradation) after HU. Cells were incubated successively with CldU (red) and IdU (green) for 20 min each and then exposed to 4 mM HU for 4 h. Bottom: dot plot of IdU/CldU ratio and median (red lines) from U-2 OS (WT) and SCAI-KD cells transfected with siRNA against BRCA2 (data combined from n = 2 with similar results). (D) Similar experiment as in (B) but from cells transfected with siNT or siRNA against BRCA2 (data combined from n = 2 with similar results). (E) UV sensitivity of SCAI-KO (#1) cells is not rescued by siRNA-mediated depletion of EXO1. Values are the mean ± SEM from 2 independent experiments. Statistics used: Kruskal–Wallis with Dunn’s multiple comparisons test (B-D). ns: nonsignificant, ***: p ≤ 0.001 ****: p ≤ 0.0001. The data underlying the graphs shown in the figure can be found in S1 Data. CldU, 5-chloro-2′-deoxyuridine; HU, hydroxyurea; IdU, 5-iodo-2′-deoxyuridine; KD, knockdown; KO, knockout; RF, replication fork; SEM, standard error of the mean; WT, wild type. (TIF) [file pbio.3001543.s005.tif]

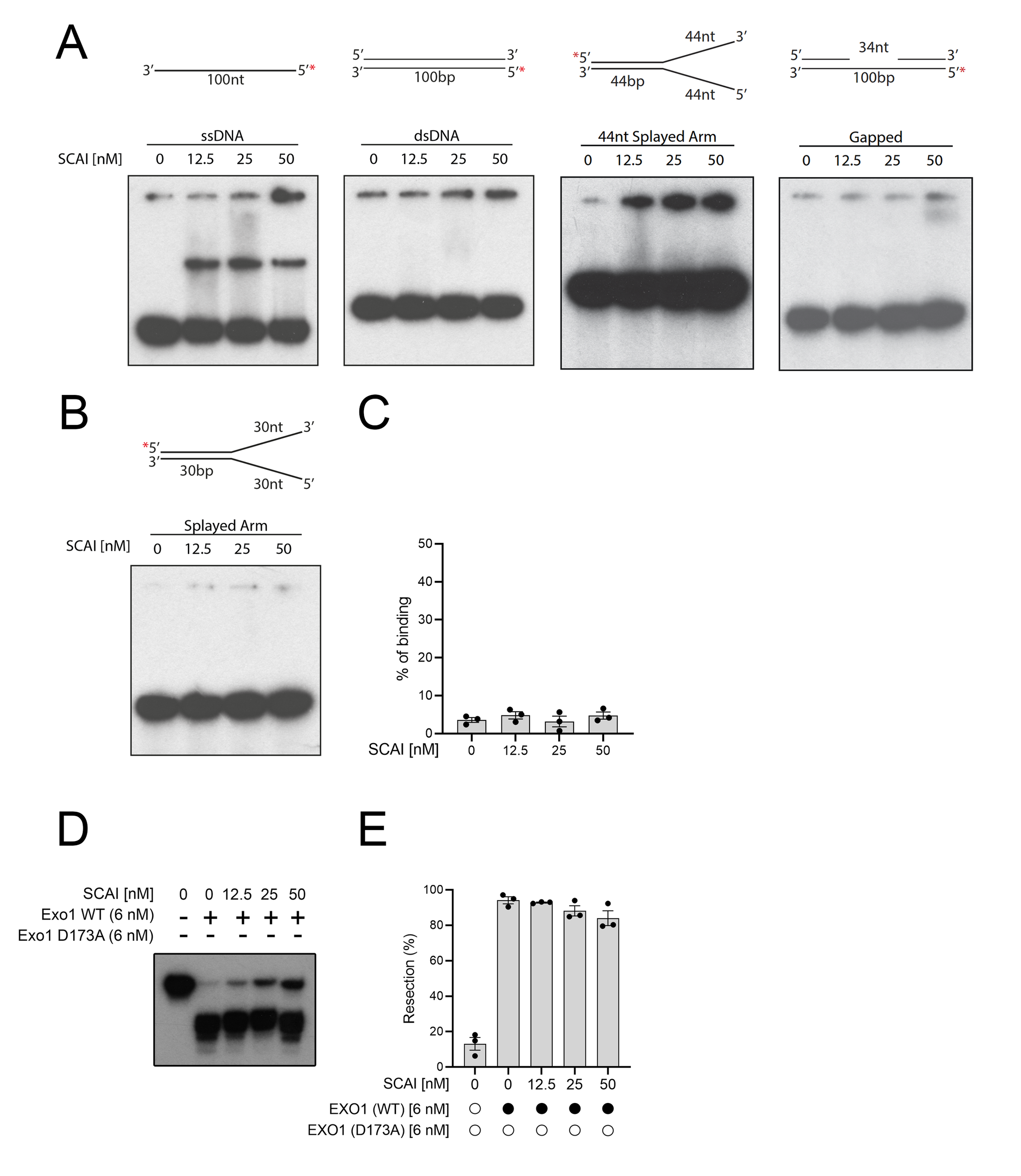

Supplement: S6 Fig — (A) 5′-[32P]-labeled ssDNA, dsDNA, splayed arm (44 nt arm length), or gapped DNA were incubated with purified recombinant SCAI at increasing concentrations and the reaction products separated by acrylamide gel electrophoresis and visualized using autoradiography. (B) Similar experiment as in (A) but using a splayed arm DNA substrate with 30 nt arm length. (C) Quantification of SCAI binding from (B). (D) In vitro DNA resection assays using the 5′-[32P]-labeled splayed arm DNA substrate (44 nt arm length) in the absence of any proteins, with WT or a catalytically inactive version of EXO1 (D173A) supplemented with purified recombinant SCAI. (E) Quantification of the percentage of DNA resection from (D). Cartoons of the various substrates are shown on top of their respective gel. Autoradiographs are representative results from 3 independent experiments. The data underlying the graphs shown in the figure can be found in S1 Data. dsDNA, double-stranded DNA ssDNA, single-stranded DNA; WT, wild type. (TIF) [file pbio.3001543.s006.tif]

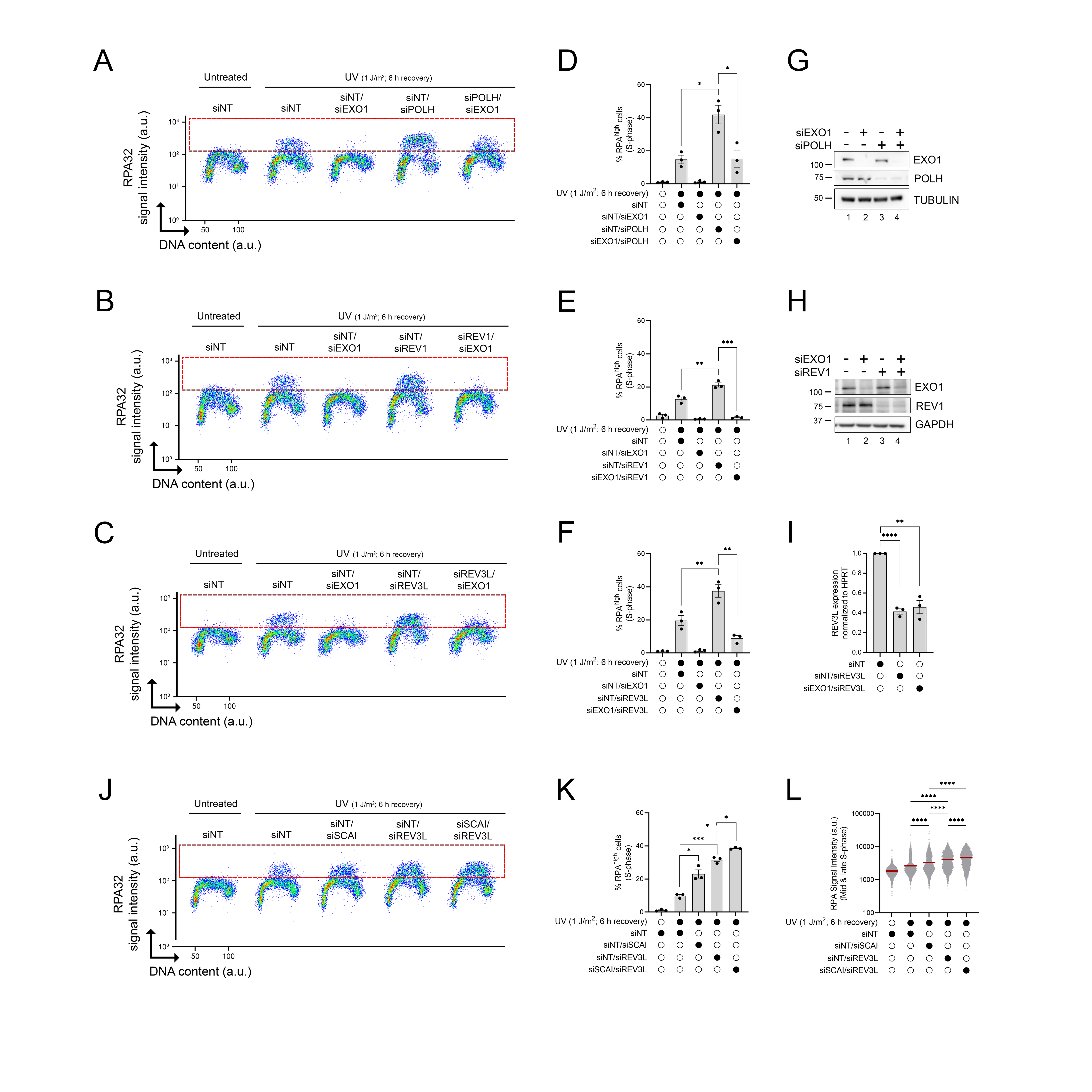

Supplement: S7 Fig — (A-C) Depletion of EXO1 rescues DNA-bound RPA formation in cells lacking Polη (A), REV1 (B), or REV3L (C) post-UV. Representative immunofluorescence flow cytometry plots used to measure RPA32 (y-axis) and total DNA content (x-axis; DAPI signal). Cells were treated with 1 J/m2 UV or mock-treated and samples were collected 6 h post-UV. The dashed red box delineates DNA-bound RPAhigh cells. (D-F) Quantification from (A-C). (G-I) KD efficiency of siRNAs from (A-C) was confirmed by immunoblotting (G-H) or RT-qPCR (I). (J-L) Depletion of SCAI increases the level of DNA-bound RPA32 in cells lacking REV3L. Representative immunofluorescence flow cytometry plots were used to measure RPA32 (y-axis) and total DNA content (x-axis; DAPI signal). Cells were treated with 1 J/m2 UV or mock-treated, and samples were collected 6 h post-UV. The dashed red box delineates DNA-bound RPAhigh cells. (K) Quantification from (J). (L) Representative quantification of RPA32 signal intensity from cells in mid and late S phase. Lines represent the mean. Values in bar graphs represent the mean ± SEM from 3 independent experiments. Statistics used: unpaired t test corrected for multiple comparisons using the Holm–Šídák method (D-F, I, and K), one-way ANOVA corrected for multiple comparisons using the Tukey method (L). *: p ≤ 0.05, **: p ≤ 0.01, ***: p ≤ 0.001, ****: p ≤ 0.0001. The data underlying the graphs shown in the figure can be found in S1 Data. a.u., arbitrary units; KD, knockdown; RPA, Replication Protein A; RT-qPCR, quantitative real-time PCR; SEM, standard error of the mean ssDNA, single-stranded DNA; TLS, translesion synthesis; (TIF) [file pbio.3001543.s007.tif]
