# Supplementary material for: A genome-wide screen identifies SCAI as a modulator of the UV-induced replicative stress response
Source: PLoS Biol. 2022 Oct 10;20(10):e3001543. doi: 10.1371/journal.pbio.3001543 (PMC9584372; doi:10.1371/journal.pbio.3001543)

Raw images for Fig 2B

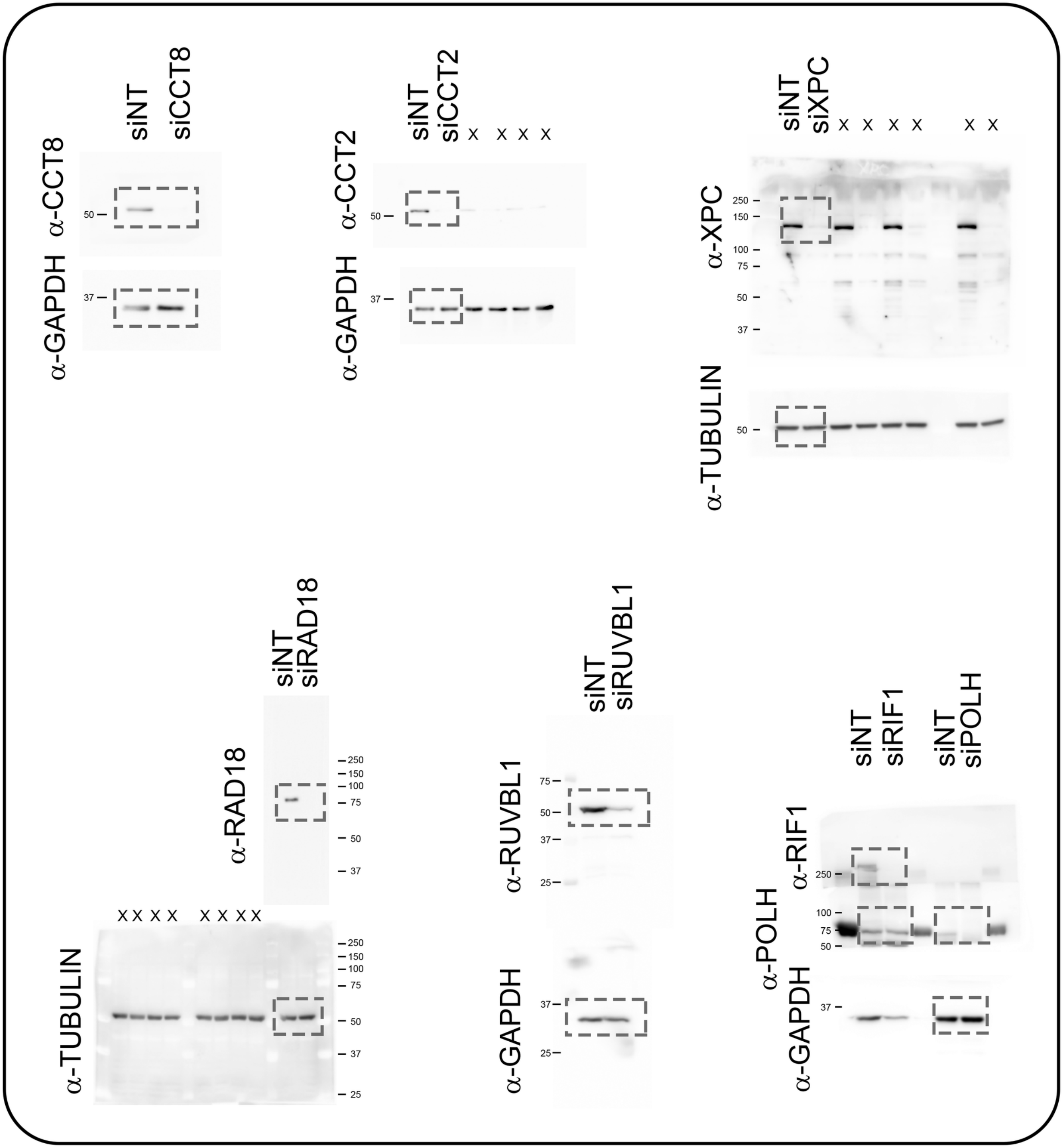

Raw images for Fig 3

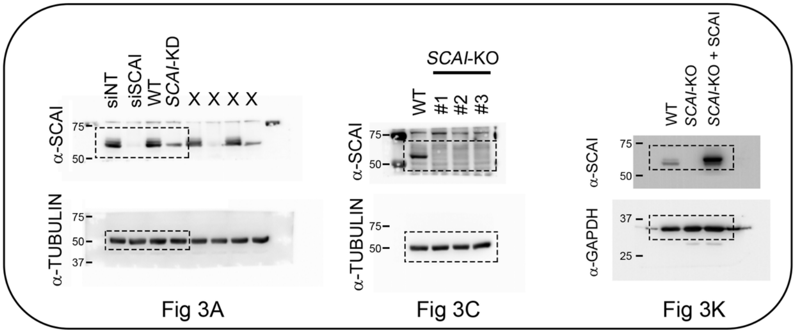

Raw images for Fig 4

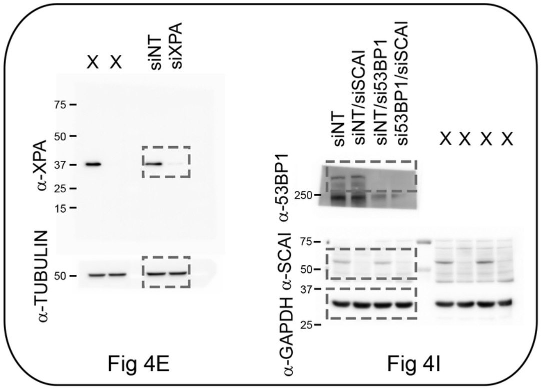

Raw images for Fig 5

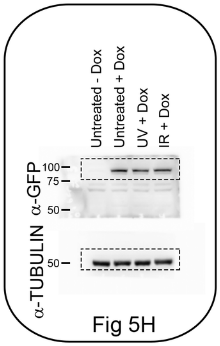

Raw images for Fig 6

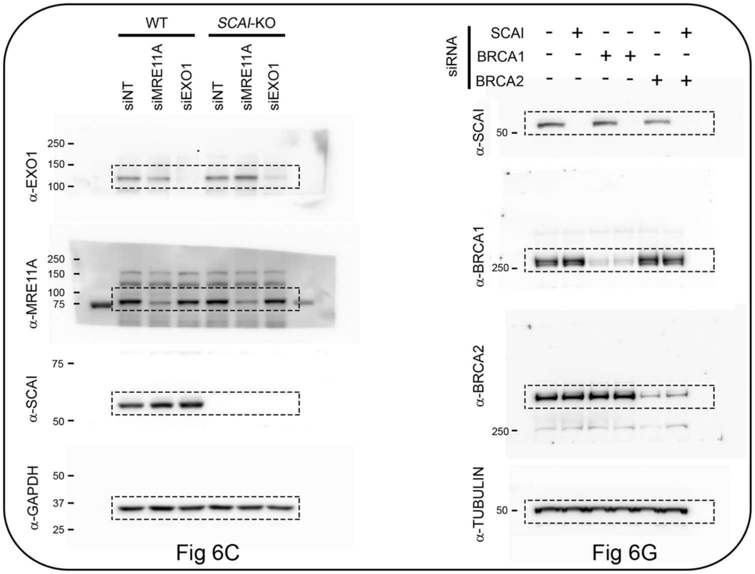

Raw images for Fig 7

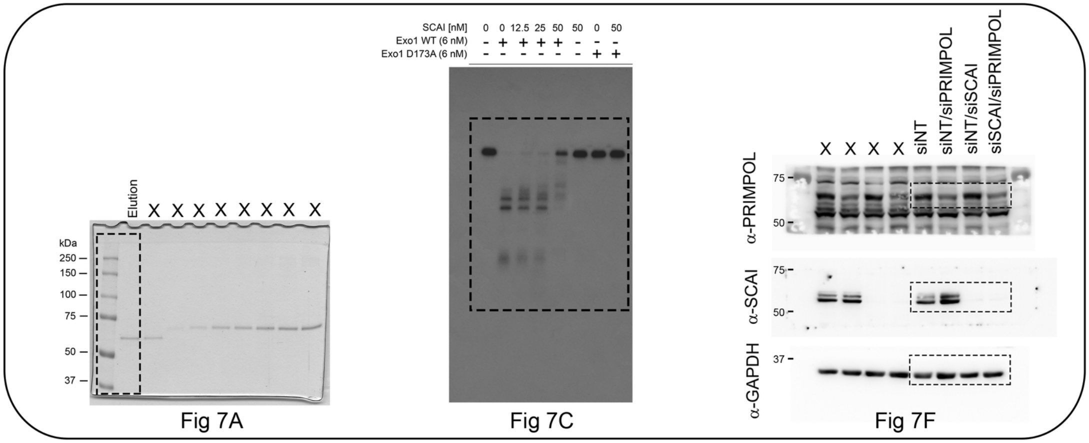

Raw images for Fig S1

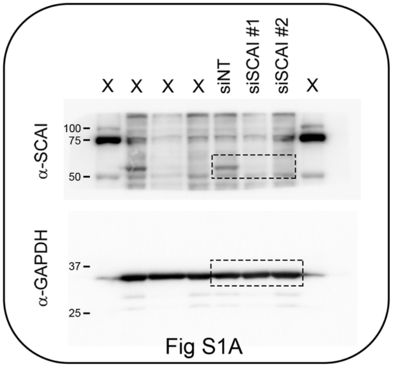

Raw images for Fig S2

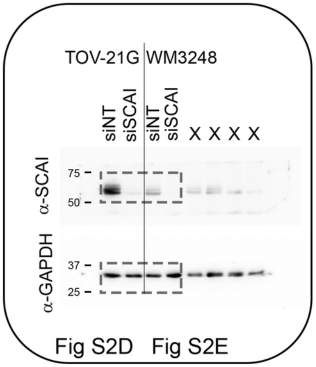

Raw images for Fig S6

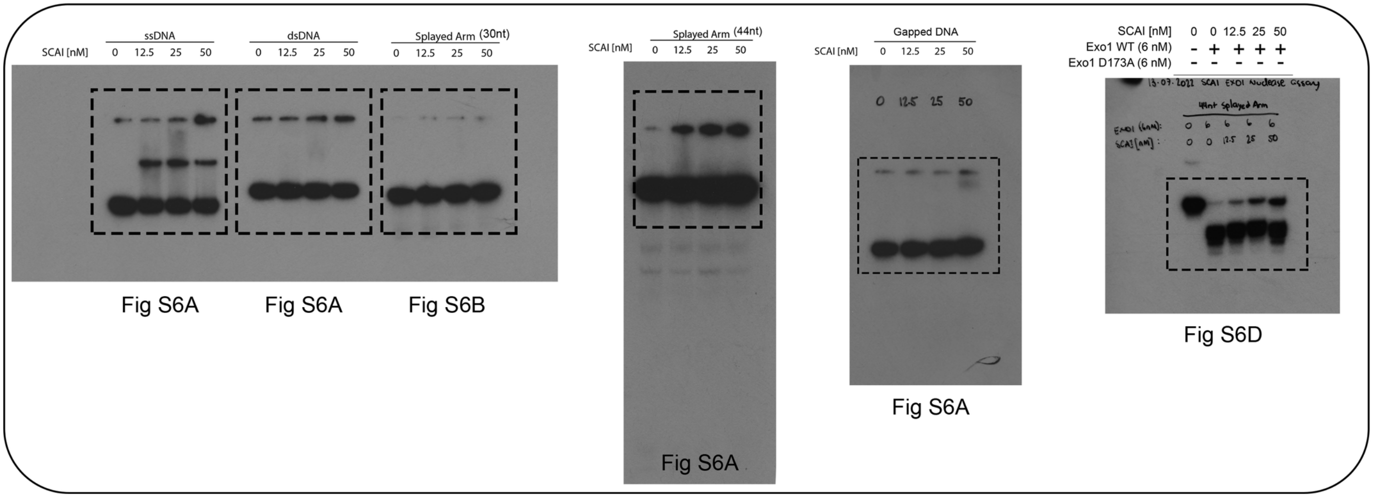

Raw images for Fig S7

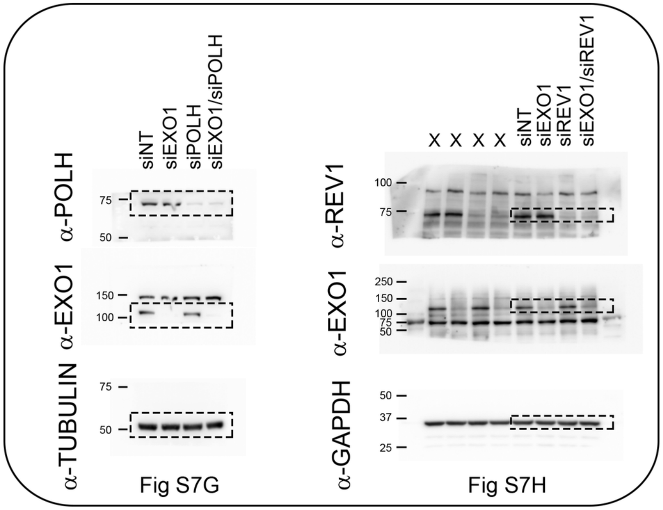

Supplement: S1 Raw Images — (PDF) [file pbio.3001543.s009.pdf]
